# Comparing biotic drivers of litter breakdown across stream compartments

**DOI:** 10.1111/1365-2656.13000

**Published:** 2019-05-17

**Authors:** Ignacio Peralta‐Maraver, Daniel M. Perkins, Murray S. A. Thompson, Katarina Fussmann, Julia Reiss, Anne L. Robertson

**Affiliations:** ^1^ Department of Life Sciences Roehampton University London UK; ^2^ Centre for Environment, Fisheries and Aquaculture Science Lowestoft Laboratory Suffolk UK

**Keywords:** benthos, hyporheos, litter breakdown, multivariable mediation models, nutrients processing, streambed ecology

## Abstract

1. Litter breakdown in the streambed is an important pathway in organic carbon cycling and energy transfer in the biosphere that is mediated by a wide range of streambed organisms. However, most research on litter breakdown to date has focused on a small fraction of the taxa that drive it (e.g. microbial vs. macroinvertebrate‐mediated breakdown) and has been limited to the benthic zone (BZ). Despite the importance of the hyporheic zone (HZ) as a bioreactor, little is known about what, or who, mediates litter breakdown in this compartment and whether breakdown rates differ between the BZ and HZ.
2. Here, we explore the relationship between litter breakdown and the variation in community structure of benthic and hyporheic communities by deploying two standardized bioassays (cotton strips and two types of commercially available tea bags) in 30 UK streams that encompass a range of environmental conditions. Then, we modelled these assays as a response of the streambed compartment and the biological features of the streambed assemblage (Prokaryota, Protozoa and Eumetazoa invertebrates) to understand the generality and efficiency of litter processing across communities.
3. Litter breakdown was much faster in the BZ compared with the HZ (around 5 times higher for cotton strips and 1.5 times faster for the tea leaves). However, differences in litter breakdown between the BZ and the HZ were mediated by the biological features of the benthos and the hyporheos. Biomass of all the studied biotic groups, α‐diversity of Eumetazoa invertebrates and metabolic diversity of Prokaryota were important predictors that were positively related to breakdown coefficients demonstrating their importance in the functioning of the streambed ecosystem.
4. Our study uses a novel multimetric bioassay that is able to disentangle the contribution by Prokaryota, Protozoa and Eumetazoa invertebrates to litter breakdown. In doing so, our study reveals new insights into how organic matter decomposition is partitioned across biota and streambed compartments.

## Graphical abstract

The authors use a novel multimetric bioassay that is able to disentangle the contribution by Prokaryota, Protozoa and eumetazoan invertebrates to litter breakdown. The study reveals new insights into how organic matter breakdown is partitioned across biota and streambed compartments.

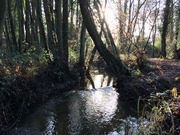

## 1 INTRODUCTION

Globally, terrestrial plants produce approximately 120 billion tons of organic carbon annually (Beer et al., 2010) and more than 90% of this production escapes from herbivores (Gessner et al., 2010). Thus, the breakdown of plant litter is an essential biosphere‐scale ecosystem process (Boyero et al., 2011; Datry et al., 2018). Despite covering less than 1% of the Earth's surface, streams and rivers contribute significantly to litter processing, and by extension to the global carbon cycle (Battin et al., 2008, 2009; Burrows et al., 2017). Traditionally, this ecological process is assessed with leaf litter assays in streambed studies (Webster & Benefield, 1986). However, this approach presents considerable limitations as a standardized method in large‐scale studies (Tiegs, Langhans, Tockner, & Gessner, 2007). For this reason, researchers have begun to use artificial substrates, such as cotton strips (e.g. Tiegs et al., 2007; Tiegs, Clapcott, Griffiths, & Boulton, 2013; Tiegs et al., 2019) or commercial tea bags (Keuskamp, Dingemans, Lehtinen, Sarneel, & Hefting, 2013), to obtain standardized global‐scale breakdown data.

The rate of litter breakdown in the streambed depends on a multivariable and sequential process, including the dissolution of labile compounds (leaching), microbial conditioning, consumption, fragmentation and environmental abrasion (Webster & Benfield, 1986). Thus, factors driving litter breakdown in streams include both abiotic and biotic components (Webster & Benefield, 1986). In addition, many of the biological processes that drive leaf litter breakdown are constrained by the environment, for example, temperature, pH and oxygen (Gessner, Chauvet, & Dobson, 1999; McArthur, Barnes, Hansen, & Leff, 1988; Thompson & Bärlocher, 1989). During this process, many taxonomic groups are involved: prokaryotic (Archaea and Bacteria) and fungal consortia drive the initial litter decomposition processes (Gulis & Suberkropp, 2003); then, Protozoa (including ciliates and flagellates) inhabiting the sediment pore space might stimulate prokaryotic population growth and activity (Peralta‐Maraver, Galloway, et al., 2018; Risse‐Buhl et al., 2012). Using microcosms, Ribblett, Palmer, and Wayne Coats (2005) found that the decay coefficient of leaf litter was up to three to four times higher when bacterivorous Protozoa were present compared with treatments in which they were excluded (Ribblett et al., 2005). This initial microbial processing, so‐called leaf‐conditioning, increases the palatability and quality of leaf litter as a food resource for invertebrate shredders (Abelho, 2008; Foucreau, Piscart, Puijalon, & Hervant, 2016; Gonçalves et al., 2017). In addition to direct consumption, Eumetazoa invertebrates might also intervene indirectly in the breakdown process. Life activities of these organisms (e.g. chironomid species, oligochaeta species) produce bioturbation and bioirrigation phenomena in the streambed (Baranov, Lewandowski, & Krause, 2016; Baranov, Lewandowski, Romeijn, Singer, & Krause, 2016; Mermillod‐Blondin & Rosenberg, 2006), which could enhance prokaryotic activity and oxidation of organic matter (Baranov, Lewandowski, Romeijn, et al., 2016; Kristensen et al., 2012). Thus, it can be expected that a wide range of taxa inhabiting the streambed, not just biofilms and shredders, might be involved in litter breakdown in some way. Yet, studies that are able to distinguish between different components of the streambed assemblage beyond microbes and macroinvertebrates are rare (Peralta‐Maraver, Galloway, et al., 2018; Reiss & Schmid‐Araya, 2008, 2010). Little is known of how Prokaryota, Protozoa and Eumetazoa invertebrates mediate breakdown rates and how this is affected by environmental conditions (Boulton, 2000; Cornut, Elger, Lambrigot, Marmonier, & Chauvet, 2010; Marmonier et al., 2012; Navel et al., 2010; Peralta‐Maraver, Galloway, et al., 2018).

The streambed can be compartmentalized into two vertical zones, the benthic zone (BZ) and the hyporheic zone (HZ). The BZ is broadly located in the upper 10 centimetres of the streambed (e.g. Smock, Gladden, Riekenberg, Smith, & Black, 1992; Reynolds & Benke, 2012, Peralta‐Maraver, Galloway, et al., 2018), in direct contact with the stream water flow and exposed to light. The HZ encompasses the volume of sediment beneath the BZ where surface water interacts with groundwater (Battin, Besemer, Bengtsson, Romani, & Packmann, 2016; Boulton, Findlay, Marmonier, Stanley, & Valett, 1998; Findlay, 1995; Robertson & Wood, 2010). Both compartments are certainty distinct environments with characteristic abiotic conditions (Peralta‐Maraver, Galloway, et al., 2018; Peralta‐Maraver, Galloway, et al., 2018), and it is likely that litter breakdown differs between these zones. The majority of leaves falling into streams and rivers are trapped by streambed structures, mostly cobbles and woody debris, forming leaf packs that are processed in the BZ (Cummins, Petersen, Howard, Wuycheck, & Holt, 1973; Peralta–Maraver, 2011). However, a substantial part of the total leaf litter entering streams and rivers is buried and stored in the HZ as a consequence of storm events, flooding and sediment movements (Cornut et al., 2010). Once in the HZ, gross litter breakdown seems to be markedly reduced in comparison with the BZ (Cornut et al., 2010; Danger, Cornut, Elger, & Chauvet, 2012). Furthermore, both streambed compartments house discrete biological communities, which quantitatively differ in composition and relative abundance of taxa (Peralta‐Maraver, Galloway, et al., 2018). Therefore, it seems likely that the benthic (benthos) and hyporheic (hyporheos) communities might also differ in their ability to process leaf litter although this has not yet been evaluated. Additionally, the links between streambed compartmentalization (BZ vs. HZ), assemblages of interstitial organisms (including benthos and hyporheos), prokaryotic metabolic activity and litter breakdown in the streambed are also poorly studied. Thus, large‐scale studies incorporating the variables described above will notably improve the current understanding of this important ecosystem process.

Here, we investigate the main biological factors driving the rate of litter breakdown in the streambed following a regional‐scale approach and involving a large range of taxonomic groups (Prokaryota, Protozoa and Eumetazoa invertebrates). Firstly, we carried out a large survey to study the relationship between litter breakdown and the variation in community structure (composition and relative abundance of taxa) of benthos and hyporheos across 30 UK streams. Subsequently, we explored the environmental and biological variables behind this relationship, and how they differ between streambed compartments, in order to identify the main mediators of litter breakdown. For this purpose, biomass and α‐diversity of Protozoa and Eumetazoa invertebrates, as well as biomass, potential metabolic activity and metabolic diversity of Prokaryota, were included in our analyses. Finally, based on the knowledge acquired during these descriptive stages and under a framework of mediation analysis, we built predictive models for the decay coefficients of the different substrata and stabilization factor (S, the proportion of leaf litter that escapes from processing and becomes recalcitrant as a consequence of environmental factors; Keuskamp et al., 2013) as responses of the identified drivers behind the process. With the obtained models, we tested the following hypotheses:

1. BZ is the most active part of the streambed, and therefore, decay coefficients are higher here than in the HZ, while on the contrary S is reduced. Consequently, the streambed compartment is an important predictive factor of litter breakdown.
2. All the taxonomic components of the streambed assemblage are important gears during litter breakdown. Thus, biomass and α‐diversity of Protozoa and Eumetazoa invertebrates, as well as biomass, and metabolic diversity of Prokaryota will be detected as significant predictors in our inferential models with a positive effect on the responses, demonstrating the necessity of including all taxonomic groups when assessing ecosystem processes in the streambed.
3. The direct effect of zone on decay coefficients and S has a lower explanatory power regarding litter breakdown variability than its mediated effect through biological features that differ between BZ and HZ.

## 2 MATERIALS AND METHODS

### 2.1 Survey design and sample processing

**Figure 1:**
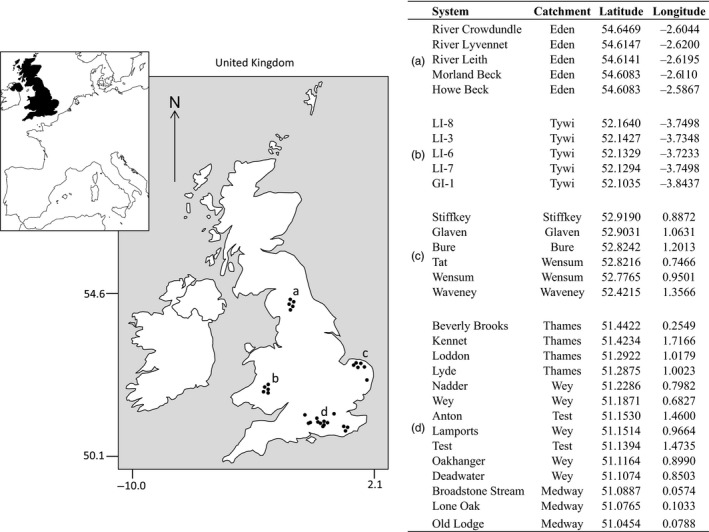
Locations of the study systems in the United Kingdom including the catchment area to which they belong, latitude and longitude

Breakdown rates were assessed in the BZ and the HZ of 30 streams from 10 different catchments located across England and Wales in the UK (Figure 1). Streams ranged from small upland, acidic headwaters to large lowland, base‐rich chalk streams, which allowed us to relate changes in decomposition with biotic and environmental gradients. Canopy cover, sediment morphology (cobbles, gravel, sand and silt) and the quantity of leaf litter, submerged plants and submerged wood were characterized semi‐quantitatively in situ at each site (giving values ranging from 0 when none were present to a maximum of 3). Meanwhile, measurements of pH, altitude, latitude, longitude, dissolved organic carbon, dissolved inorganic nitrogen, ammonium, nitrate and phosphate were obtained from the UK Environment Agency as annual averages when available. A detailed characterization of the study sites is available as Table S1.

The cotton‐strip assay and the tea bag index (TBI) were applied to measure litter breakdown. The cotton‐strip assay has been widely used as a readily standardized test to measure litter breakdown in rivers and riparian sites around the world (e.g. Tiegs et al., 2019). This method is based on the loss of tensile strength of cotton strips after a certain incubation period in the field (Tiegs et al., 2007). The TBI is a recent approach applied in the terrestrial environment which uses commercially available tea bags as highly standardized test kits to measure litter breakdown (Keuskamp et al., 2013). It uses two types of tea with contrasting decomposability (green tea and rooibos tea), allowing the separate measurement of the decay coefficients of each tea type based on loss in weight after an incubation period. The acquired TBI consists of two parameters describing global litter decay coefficient (K) and long‐term litter stabilization factor (S; Keuskamp et al., 2013). This method also allows the separate measurement of the decay coefficients of each tea type. In this study, sampling units consisted of a coarse mesh package (mesh size = 0.5 cm; henceforth bioassay) containing the three different organic substrates: two types of tea within tetrahedron‐shaped synthetic tea bags (Lipton; mesh size = 0.25 mm) and a single cotton strip (8.0 cm × 2.0 cm; made of 100% unbleached cotton, 96% cellulose).

The mesh packages were fixed in pairs with a rope to an iron rod in the streambed sediments; one rested on the streambed at 0–2 cm depth within the sediment (BZ) and the other at 15 cm (HZ) depth. Deployment began on 25 October 2016 during peak leaf fall and was completed within 61 days. Three pairs of packages were deployed along a 50‐m section per stream (3 repeat measures per zone per stream, 180 packages in total). A piezometer pipe with a conical tip fitted on the bottom was used to bury the HZ bioassays. At each stream, a temperature data logger (iButton DS1922L, accuracy of ±0.5°C) was attached to one of the mesh packages at 15 cm depth, which recorded temperature every 10 min in the HZ. Spot surface‐water temperature measurements were recorded during collection of samples and the exact time was recorded, so that it was possible to compare them with the equivalent hyporheic values. Afterwards, a simple linear regression of surface temperature as response of the equivalent time measurements in the HZ was applied to infer variation of temperature in the BZ during the study period (regression coefficients and model visualization are available as Figure S1). Despite hydraulic retention in the hyporheic zone might buffer the daily variation in surface‐water temperature through depth (Arrigoni et al., 2008), we assumed small differences in the vertical profile when comparing 2‐ and 15‐cm‐depth layers (e.g. Conant, 2004; Peralta‐Maraver, Galloway, et al., 2018).

Bioassays were deployed for between 29 and 61 days (high flows delayed planned bioassay retrieval for some sites meaning incubation time differed between streams; see Table S1). Bioassays were carefully removed from the BZ and HZ using a gardener hand trowel, kept in 50‐ml falcon vials filled with autoclaved mineral water, returned to the laboratory using an ice‐chilled cooler and processed within 48 hr of collection. To account for any differences in bioassay volume, which could confound estimates of unit volume biomass, each bioassay's volume was determined as the difference between the total vial volume and the added water volume (volume ranged between 38 and 45 ml). Once in the laboratory, water subsamples from the falcon vials were extracted for Prokaryote and Protozoa processing, while the remaining content of the vials was retained on a 40‐µm sieve for Eumetazoa invertebrate processing (see Methods S1).

Tea bags were dried at 60°C for 48 hr, and dry weight of content was measured using an electric scale (accuracy of 0.1 µg). Breakdown coefficients were calculated following Keuskamp et al. (2013). Cotton strips were soaked in 70% ethanol to inhibit microbial activity during storage, then air‐dried and stored individually in paper envelopes. Following Tiegs et al. (2007), tensile strength of all cotton strips was measured (preparation and processing of cotton strips and tea bags, including initial tensile strength and weight, is available as Methods S1). Following Woodward et al. (2012), breakdown rates were expressed as the exponential decay coefficient (*k*) in the formula:

$$X_{t}/X_{0}=e^{-kt}$$

where *X*_0_ is the initial leaf mass or tensile strength, and *X_t_* is the value upon removal of the bioassays from the field at time *t*. The exponential coefficient *t* was expressed in terms of thermal sums (degree‐days) to correct for potential temperature effects and/ or differences in days of deployment (exponential decay model is a good fit with the available time‐series data for the tea bags; Figure S2).

We examined breakdown between different size groups of organisms using the differing mesh sizes separating the organic substrates in the bioassay. Cotton strips were accessible to all organisms in the assemblage below 0.5 cm (i.e. bioassay outer mesh size). The tea bag mesh size of 0.25 mm allowed mostly micro‐organisms (Prokaryota and Protozoa) to enter the tea bags, excluding all but the very smallest Eumetazoa invertebrates.

### 2.2 Biomass and diversity of Protozoa and invertebrates

Protozoa, including ciliates and flagellates, from the stored unfiltered water were identified and counted alive under an Olympus BX50 Microscope. Ciliates subsamples were processed using a Sedgewick Rafter counting cell chamber (1 ml volume; Pyser‐SGI Limited, Edenbridge, UK), while flagellates were processed using a Neubauer cell counting chamber. Ciliates were identified to subclass using identification keys (Foissner & Berger, 1996), while flagellates were treated as a single group. Eumetazoa invertebrates were extracted from the preserved subsamples under a Nikon SMZ‐U Stereomicroscope (30x), identified to species level in most of the groups (Table S2) using identification keys (Rundle, Robertson, & Schmid‐Araya, 2002; Tachet, Richoux, Bournaud, & Usseglio‐Polatera, 2002) and counted. Following Peralta‐Maraver, Galloway, et al. (2018), length and width of all counted Protozoa and Eumetazoa invertebrates were measured to the nearest micrometre and converted in dry carbon content (see also Supporting Information Material: Methods). Lastly, biomass (mg C/L) of all identified taxa was obtained by multiplying dry carbon content (mg C) with individual density (ind/L).

Protozoa and Eumetazoa invertebrates’ α‐diversity (Shannon–Wiener diversity) was estimated by setting a base‐sample size and using rarefaction and extrapolation based on Hill numbers following Hsieh, Ma, and Chao (2014). This approach is considered a robust method for comparing diversity between communities where sample sizes differ (Chao et al., 2014). Furthermore, it solved related collinearity problems between diversity and biomass. In this manner, both variables could be incorporated in our analytical models (see below). Calculations of α‐diversity were made using the R package iNEXT (R Core Team, 2018; Hsieh et al., 2014).

### 2.3 Biomass and potential metabolic activity of Prokaryota

Prokaryotic biomass was assessed after cell counting in the filtered stored water. To stain the DNA of living cells, 200 μl of PicoGreen dye solution (Quant‐iT™ PicoGreen™ dsDNA Assay Kit, Sigma‐Aldrich) was added to 1 ml filtered water and incubated at 4°C for 15 min. Prokaryotic cell counts were then measured using an Accuri C6 flow cytometer (BD Biosciences) on slow with a forward scatter‐H of 8000 and a side scatter‐H of 2000. The list of individual events returned by the flow cytometer was extracted using the R packages flowCore and flowViz (Ellis, Haaland, Hahne, Le Meur, & Gopalakrishnan, 2009; Sarkar, Meur, & Gentleman, 2008). Next, following Schaum et al. (2017) individual cell sizes were estimated using calibration beads to convert forward scatter to average diameter of bacterial cells (Figure S3), and biomass values were inferred from published relationships between cell size and carbon content (Fuhrman & Azam, 1980; Watson, Novitsky, Quinby, & Valois, 1977).

Prokaryota potential activity (as aerobic metabolic potential to utilize different carbon sources) was measured by incubating filtered water subsamples in Biolog EcoPlate Systems (Biolog Inc.). Following Feigl, Ujaczki, Vaszita, and Molnár (2017), changes in the coloration of tetrazolium violet redox dye were used to measure prokaryotic respiration, and prokaryotic metabolic diversity was calculated as Shannon–Wiener diversity value (H') based on substrate utilization (see also Methods S1).

### 2.4 Statistical analysis

We first applied a NMDS ordination model based on Bray–Curtis index (Oksanen et al., 2013) to compare the dissimilarities in community structure (composition and relative abundance of taxa) between compartments and across the 30 studied rivers. Information from the three collected bioassays was pooled by compartment (BZ and HZ) and studied river prior to the ordination. Subsequently, environmental gradients (Table S1), biological descriptors of the community (biomass, α‐diversity, prokaryotic potential metabolic activity and prokaryotic metabolic diversity) and streambed compartment (two‐level factors: BZ and HZ) were fitted to the ordination. The degree of association between these variables and the ordination was assessed by comparing the model of pairwise interactions with 1,000 permutations of a given null model. Secondly, we applied one‐way ANOVA tests to characterize differences in the measured biological variables (response variables) between streambed compartments (predictor variable).

We detected a high correlation between streambed compartment and the studied biological variables. Therefore, we adopted a framework of mediation regression analysis to test whether the biological variables significantly associated with the NMDS ordination mediate the effect of streambed compartment on the decay coefficients of green tea (*k*_green_), red tea (*k*_red_), cotton strips (*k*_cotton_), global litter decay coefficient (*K*) and long‐term carbon stabilization factor (*S*). Within this framework of mediation analysis, the total effect of an intervention on an outcome variable is decomposed into a direct and indirect effect (MacKinnon, 2012; Rijnhart, Twisk, Chinapaw, de Boer, & Heymans, 2017; Figure S4). In our study, the indirect effect goes through the measured biological variables, and the remaining effect reflects the direct effect of streambed compartments on decay coefficients and S. After model selection routines, optimal mediated regression models were fitted as part of the causal three‐step method proposed by Judd and Kenny (1981) for statistical mediation analysis (see Methods S1).

Each bioassay collected from the studied systems was treated as the sampling unit in the ANOVA tests and the predictive models. Therefore, study site (30 levels) and catchment (10 levels) were incorporated in the ANOVA tests as nested random factors (random‐factors ANOVA) and the mediated regression models as random intercepts (linear mixed models, LMMs). Thus, it was possible to deal with the non‐independence of repeated measurements per study site and the nested intraclass correlation effects of river and catchment.

The NMDS ordination and the subsequent variable fitting were carried out with the metaMDS and envfit functions of the r package vegan (Oksanen et al., 2013). ANOVA tests and mediated LMMs were fitted using the restricted maximum likelihood estimation (REML) with the lmer function of the r package lme4 (Bates, Maechler, Bolker, & Walker, 2014). A detailed explanation of the models selection routines, models fitting and models validation is available in the Methods S1.

## 3 RESULTS

**Figure 2:**
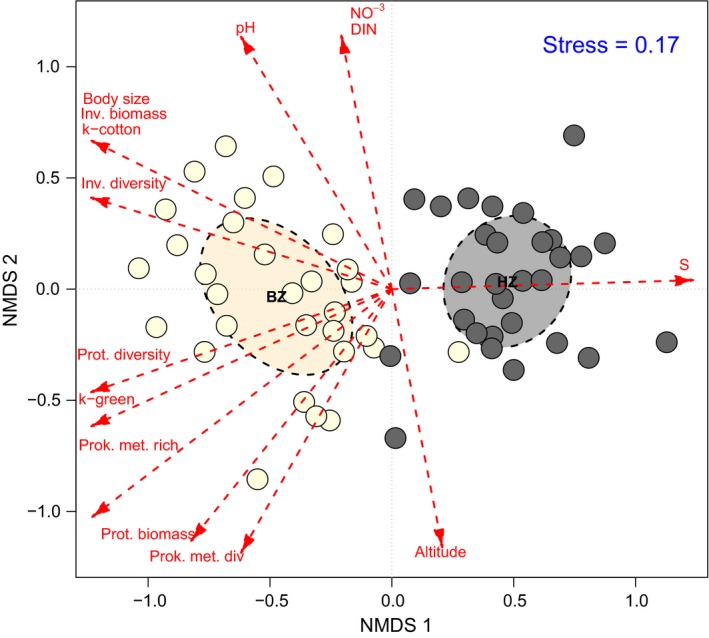
NMDS ordination model based on Bray–Curtis index comparing the dissimilarities in composition and abundance of benthos (yellow dots) and hyporheos (grey dots) across the 30 studied systems. Ellipses show the 95% CIs on the location of centroids. Environmental gradients, biological descriptors of the communities and breakdown coefficients that were significantly correlated (*p* < 0.05) with the ordination are overlapped with the ordination. The arrows depict the relationship of fitted variables with the ordination (NO_3_ = nitrate; DIN = dissolved inorganic nitrogen; Inv. biomass = invertebrate biomass; Prot. biomass = Protozoa biomass; Inv. diversity = Eumetazoa invertebrate diversity; Prot. diversity = Protozoa diversity; Prok. met. rich = Prokaryota metabolic richness; Prok. met. div. = Prokaryota metabolic diversity; *k*_cotton_ = decay coefficient of cotton strips; *k*_green_ = decay coefficient of green tea; S = long‐term carbon stabilization factor)

A total of 7,136 Eumetazoa invertebrates, 11,436 ciliates and 2,544 flagellates were collected and identified to measure biomass and α‐diversity giving a good representation of the streambed assemblages (the identified taxa list in BZ and HZ for the 30 studied streams is available as Table S2). The NMDS ordination based on community structure (composition and relative abundance of taxa) showed a very high goodness‐of‐fit between the distances in the ordination against the original data (linear fit *R* ^2^ = 0.97, non‐metric fit *R* ^2^ = 0.85). Accordingly, the Shepard plot of the ordination presented small scatter around the fitted line (Figure S5); thus, original dissimilarities in community structure were well preserved in the reduced number of dimensions. The NMDS ordination clearly discriminated between benthos and hyporheos as discrete communities along the axis 1 (Figure 2; influence of taxa in the ordination is available as Figure S6). This variation in community structure between streambed compartments was highly significant (*R* ^2^ = 0,553; *p* = 0.001). Benthos was characterized by a greater representation of relatively large‐bodied taxa, while small‐bodied taxa gained a greater representation in the structure of the hyporheos (high correlation between body‐size gradient and streambed zonation; Figure 2). The variation in community structure along axis 1 was also significantly related to decay coefficient of cotton strips (*k*_cotton_), decay coefficient of green tea (*k*_green_) and *S*, which in turn showed a strong dependence of the biological descriptors of the community (Figure 2). The smaller variation in community structure observed on axis 2 was more related to the environmental variables; pH, nitrate, dissolved inorganic nitrogen and altitude were detected as significant gradients behind the ordination (fitting coefficients of variables significantly related to the NMDS ordination are available as Table S3).

**Figure 3:**
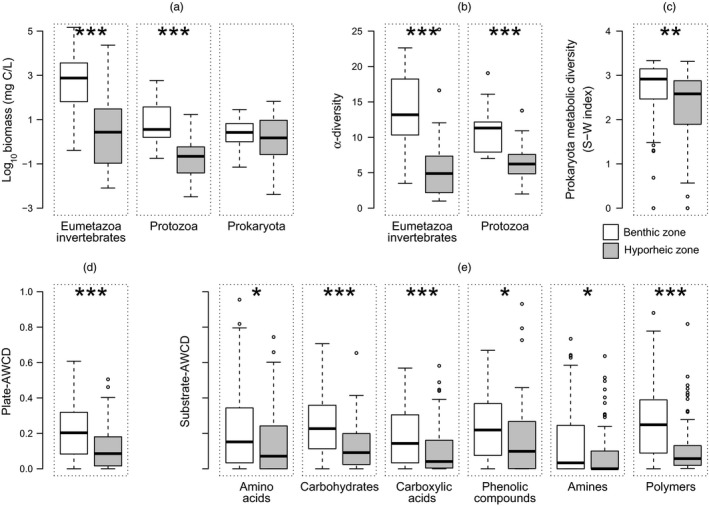
Differences between the benthic and hyporheic zone for (a) biomass of the different studied groups, (b) α‐diversity of Eumetazoa and Protozoa, (c) prokaryotic metabolic diversity, (d) plate‐AWCD and (e) substrates‐AWCD. The bold horizontal black line represents the median, whiskers show the minimum and maximum of the data, and dots represent outliers. Asterisks indicate significant differences (***p < 0.0001; **p < 0.001; *p < 0.05)

When comparing between compartments, all biological responses showed a marked decline within the HZ in comparison with the BZ, with the only exception of Prokaryota biomass (ANOVA tables including coefficients, degrees of freedom, *F*‐statistic and *p*‐values are available as Table S4). This pattern was highly significant for biomass and α‐diversity of Eumetazoa invertebrates and Protozoa (Figure 3a,b), demonstrating a great reduction and simplification of these assemblages in the HZ. Even though biomass of Prokaryota did not show any clear differences between compartments, the significantly lower metabolic potential (as plate‐AWCD and substrate‐AWCD) and metabolic diversity values illustrated the simplification in the prokaryotic assemblage within the HZ (Figure 3c,d).

Cotton strips showed the highest breakdown coefficient [*k*_cotton_; mean (*SD*) = 4.0 (3.0) × 10^‐3^], closely followed by green tea [*k*_green_, mean (*SD*) = 3.0 (0.6) × 10^‐3^], and finally rooibos tea [*k*_red_, mean (*SD*) = 0.7 (0.1) × 10^‐3^] as the more recalcitrant substratum. After model selection routines and verification of model assumptions, we were able to build suitable predictive mediation models for decay coefficients of cotton strips, green tea and rooibos tea, and for the *S* (obtained after applying TBI). In contrast, global leaf litter decay coefficient (*K*, obtained after applying TBI) did not show any clear relationship with the streambed compartment (Figure S7) or the biological covariates, neither was it possible to propose an appropriate predictive model for this response (mediated model equations and summary tables including results of the causal three‐step method, global and partial coefficients of determination, standardized coefficients, degrees of freedom, 95% credible intervals, *t*‐statistic and *p*‐values are available as Tables S5–S8). [Correction added after online publication on 15 July 2019: The units k_cotton_ (x10^3^), k_green_ (x10^3^) and k_red_ (x10^4^), have been corrected to k_cotton_ (x10^‐3^), k_green_ (x10^‐3^) and k_red_ (x10^‐4^)].

**Figure 4:**
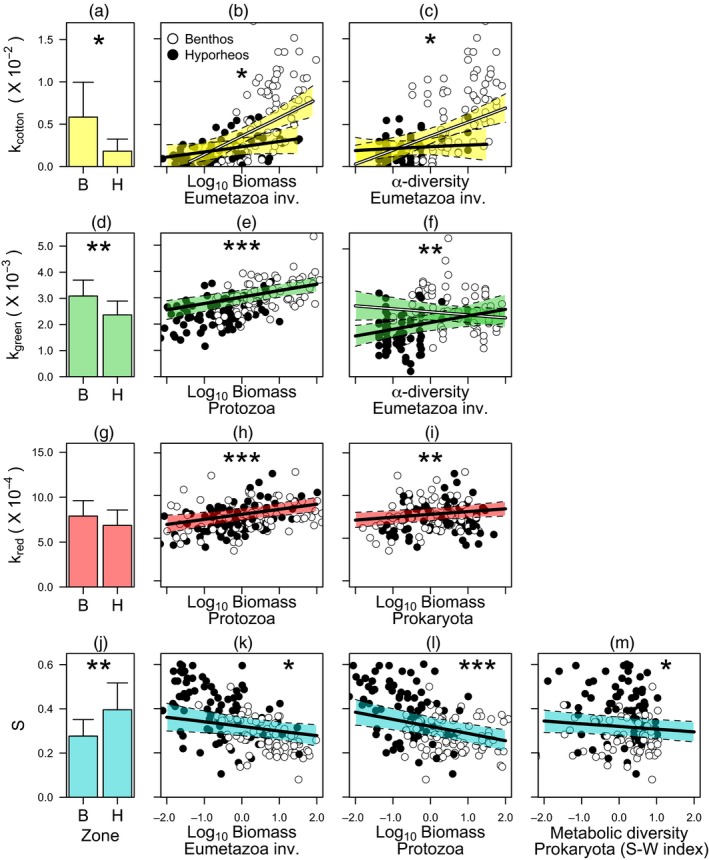
Multiple linear mixed regression models for the breakdown coefficients of cotton strips (*k*_cotton_; three top panels coloured in yellow), green tea (*k*_green_; three middle panels coloured in green), rooibos tea (*k*_red_; three bottom panels coloured in red) and long‐term carbon stabilization factor (S). Bar plots on the left show the mean value (± *SD*) of each decay coefficient in the benthic (B) and the hyporheic (H) zone. Covariates included in the models are biomass of Eumetazoa invertebrates (Biomass Eumetazoa), biomass of Protozoa (Biomass Protozoa), biomass of Prokariote (Biomass Prokariote), α‐diversity of Eumetazoa invertebrates (α‐diversity of Eumetazoa inv.), metanolic diversity of Prokaryote (Metabolic diversity Prokaryote, S‐W diversity). When interaction was significant (panels b, c and f), the black line represents fitted regression for hyporheic values and the white line represents fitted regression for benthic values. Coloured shaded areas on the regression lines represent the 95% CI of the continuous covariates. Asterisks indicate significant effect of the coefficients in the models for the *t* statistic (***p < 0.0001; **p < 0.001; *p < 0.05). Note that plot shows standardized covariates. [Correction added after online publication on 15 July 2019: The units k_cotton_ (x10^3^), k_green_ (x10^3^) and k_red_ (x10^4^), have been corrected to k_cotton_ (x10^-3^), k_green_ (x10^-3^) and k_red_ (x10^-4^)].

Total breakdown was reduced in the HZ compared to the BZ for cotton strips, green tea and rooibos tea (Figure 4a,d,g), while S reached higher values in the BZ (Figure 4j). Due to the recalcitrant nature of rooibos tea, differences in breakdown between compartments for this substrate were too weak to be detected as significant by our models. Accordingly, the BZ exhibited higher breakdown coefficients, and this was especially true for more labile substrata. Our models detected a highly significant boosting effect of biomass and α‐diversity of Eumetazoa invertebrates on breakdown of cotton strips (Figure 4b,c), biomass of Protozoa and α‐diversity of Eumetazoa invertebrates on breakdown of green and rooibos tea (Figure 4e,f) and biomass of Prokaryota on breakdown of rooibos tea (Figure 4i). In contrast, biomass of Eumetazoa invertebrates and Protozoa and Prokaryota metabolic diversity were negatively related to S (Figure 4k–m). Our models also detected a significant interaction of biomass and α‐diversity of Eumetazoa invertebrates with the streambed compartment (Figure 4b,c,f), indicating that the mediated effect is different for the two levels of the streambed compartment.

Finally, the direct effect of streambed compartment on litter breakdown was highly mediated by the studied biological variables (proportion mediated effect of 68% for cotton strips, 62% for green tea, 81% for rooibos tea and 59% for S; Tables S5–S8). Standardized coefficients of the models were always significant when computing the direct effect of streambed compartment on decay rates, S (causal three‐step method: step 1) and biological variables (causal three‐step method: step 2) (Tables S5–S8). Nevertheless, when adding the biological variables (casual three‐step method: step 3), the direct effect (standardized coefficient for compartment) decreased largely in all cases and was non‐significant for the breakdown of rooibos tea (= full mediation).

## 4 DISCUSSION

Through deploying standardized bioassays across a range of UK streams, we detected clear differences in litter breakdown between streambed compartments mediated by different organism groups. Our three hypotheses all received strong support from our study. Litter breakdown was much faster in the benthic zone compared to the hyporheic zone (roughly 5 times higher for cotton strips and 1.5 times faster for the tea leaves). As predicted, the biological features of the whole streambed assemblage were important predictors of litter breakdown and further differences in the structure of the benthos and the hyporheos were directly associated with differences in litter breakdown between streambed compartments. Especially, organismal size, identity and abundance (biomass) seem to explain the differences in decomposition rates between the zones. For example, in the benthos, the biomass of metazoans was a strong predictor of breakdown rates, but in the hyporheos, it was not – here, protozoan biomass was the strongest predictor. We found that employing three different types of organic matter was a useful approach to detect these differences. The tea bags were a good tool to access differences between the zones as in principle the same‐sized organisms (from bacteria over protozoans to microscopically small metazoans) were present in these bags and we were hence able to disentangle effects of identity and biomass on breakdown rates.

Notwithstanding the potential limitations of our sampling methodology when characterizing streambed communities (we use bioassays as colonization traps), our results strongly discriminate benthos and hyporheos as different ecological entities with individual integrity and biological characteristics. The dramatic reduction in biomass and α‐diversity of Protozoa and Eumetazoa invertebrates within the HZ in this study supports the widely reported pattern of simplification of streambed communities with increasing depth in the streambed (e.g. Schmid‐Araya, 1994; Sliva & Williams, 2005; Andrushchyshyn, Wilson, & Williams, 2007; Reynolds & Benke, 2012, Peralta‐Maraver, 2018; Dunscombe, Robertson, Peralta‐Maraver, & Shaw, 2018). Results from our regional‐scale approach are also in agreement with previous experimental assessments (Cornut et al., 2010) and local‐scale survey studies (i.e. Smith & Lake, 1993; Naamane, Chergui, & Pattee, 1999), which documented that litter breakdown is generally depressed in the HZ. Nevertheless, here we determined that differences in breakdown between the BZ and the HZ were mainly driven by differences in the biological features of the benthos and hyporheos. Biological variables explained always more than half of the effect of streambed compartmentalization (almost the total in the case of rooibos tea). Consequently, the total reduction of breakdown rates was largely explained by the simplification of community structure in the HZ, while the remaining effect of “zone” was attributable to differences in the abiotic conditions.

Our study suggests a higher litter breakdown in the BZ and a higher proportion of litter becoming recalcitrant in the HZ. It has been argued that the streambed might have a significant role in the mineralization and sequestration of organic carbon, both of which are important fluxes of the global carbon cycle (Battin et al., 2009). Our findings highlight these fluxes, but also emphasize the importance of understanding the different role of the two compartments in the streambed. Total mineralization of allochthonous organic carbon is higher in the BZ, while the HZ seems to fulfil the role of allochthonous organic carbon sink (at least for recalcitrant material). It is important to note that the litter breakdown occurs also by physical abrasion (Webster & Benfield, 1986), the extent of which might differ between compartments. Any potential difference between compartments in this respect is likely to have only minimal effects on the breakdown rates of the assays used in this study where the organic material (e.g. tea) is protected within a very fine mesh casing. Indeed, our analysis reveals that the majority of variation in breakdown rates between zones was mediated through the biological features of the assemblages.

For the first time, we report the negative effect of biomass and α‐diversity of Eumetazoa invertebrates on the stabilization of labile carbon from litter material. During litter breakdown, a proportion of the labile compounds stabilizes and becomes recalcitrant (Prescott, 2010) as a consequence of environmental factors (i.e. reacting with dissolved cations; Berg & Meentemeyer, 2002). Our results suggest that the life activities of Eumetazoa invertebrates act in opposition to the environmental influence on organic matter retention in the streambed. In the case of cotton strips, it is reasonable to advocate for direct consumption of the substratum (cotton strips were fully accessible for Eumetazoa invertebrates). Furthermore, cotton strips were a rapidly processed substratum, which could mean that extensive microbial conditioning prior to consumption by Eumetazoa invertebrates might be unnecessary (Golladay, Webster, & Benfield, 1983; Gonçalves et al., 2017). In our study, we did not consider fungal assemblages, which play a key role in the pre‐conditioning of litter material and thus its eventual consumption by invertebrates (Hieber & Gessner, 2002). Future research should include this important group and analyse differences in palatability and consumption between substrates after microbial conditioning. Tea bags, however, were not directly consumed by Eumetazoa invertebrates, and therefore, other processes became more important in determining the outcome for decay rate of green tea and S. The term Eumetazoa invertebrates includes a variety of organisms, which differ in size, shape, functional traits and movement. These diverse assemblages might undertake a variety of non‐trophic engineering phenomena, such as bioturbation and bioirrigation in the sediments (Baranov, Lewandowski, & Krause, 2016; Baranov, Lewandowski, Romeijn, et al., 2016; Mermillod‐Blondin & Rosenberg, 2006) creating an environment of high biochemical transformation and litter breakdown.

Our results revealed that an increase in biomass of Protozoa also stimulated the rate of leaf litter breakdown and reduced the proportion of sequestered labile compounds. To the best of our knowledge, this is the first field study to demonstrate the involvement of Protozoa in litter breakdown and it supports earlier laboratory results (Ribblett et al., 2005). Several potential mechanisms may explain the effect of protozoan biomass on these responses. Constant grazing on biofilms by Protozoa keeps Prokaryota consortia in the active log phase of growth (Fenchel & Jørgensen, 1977), and they usually selectively consume less active Prokaryota (Shapiro, Kushmaro, & Brenner, 2010). Thus, litter breakdown rates may increase even though the total abundance of Prokaryota decreases as a consequence of protozoan grazing (Ribblett et al., 2005). Additionally, Protozoa produce waste products that fuel the metabolism of Prokaryota (Jansson, Bergström, Blomqvist, Isaksson, & Jonsson, 1999), induce the recycling of nutrients (Shapiro et al., 2010) and increase the absorption surface of the biofilms after grazing (Peralta‐Maraver, Galloway, et al., 2018). However, controlled experiments are still necessary to test these proposed mechanisms and completely elucidate how Protozoa contribute to this ecosystem process.

The relationship of Prokaryota with litter breakdown was less obvious than for Eumetazoa invertebrates and Protozoa, but we can infer from our model for decomposition coefficient of rooibos tea (*k*_red_) that breakdown associated with microbial biomass is most important during the breakdown of recalcitrant leaf litter material. Previous studies have suggested that the contribution of Prokaryota to litter breakdown could be higher than that inferred from biomass (Findlay & Arsuffi, 1989). Here, we detected prokaryotic biomass and metabolic diversity as drivers in this process agreeing with previous research (Battin et al., 2016). Our findings state that a high metabolic diversity in prokaryotic assemblages improves the efficiency of the streambed bioreactor in processing complex litter compounds, contributing to a reduction in the environmental sequestration of labile carbon. This also supports the idea that microbial processing of plant material in aquatic realms is a major source of CO_2_ (Raymond et al., 2013).

In summary, using comparative analysis across a wide range of stream systems, carbon substrates and organismal body size, we were able to identify several key measures of ecosystem structure that predict litter processing in running waters. Our study has shown for the first time that the streambed compartment and biomass and diversity of Prokaryota, Protozoa and Eumetazoa invertebrates are important drivers of organic matter decomposition and thus play a crucial role in the wider functioning and bioreactor ability of the streambed ecosystem.

## AUTHORS' CONTRIBUTIONS

I.P.‐M., D.M.P., M.S.A.T., J.R. and A.L.R. conceived the ideas and designed the methodology; I.P.‐M., A.L.R. and M.S.A.T. carried out the sampling activities; I.P.‐M. and K.F. processed the samples; I.P.‐M. and D.M.P. analysed the data; I.P.‐M. and A.L.R. led the writing of the manuscript. All authors contributed critically to the drafts and gave final approval for publication.

## Data Availability

Data are available at the Dryad Digital Repository: https://doi.org/10.5061/dryad.55sc38s (Peralta‐Maraver, 2019).
