# Supplementary material for: Comparing biotic drivers of litter breakdown across stream compartments
Source: J Anim Ecol. 2019 May 17;88(8):1146–57. doi: 10.1111/1365-2656.13000 (PMC6851634; doi:10.1111/1365-2656.13000)
Supplement: Supplementary file 1 [file JANE-88-1146-s001.pdf]

Supplementary material for:

Comparing biotic drivers of litter breakdown across streams compartments

Ignacio Peralta–Maraver, Daniel M. Perkins, Murray S. A. Thompson, Katarina Fussmann, Julia Reiss, Anne L. Robertson.

Index

Table S1……2
Table S2……4
Table S3……7
Table S4……8
Table S5……9
Table S6……10
Table S7……11
Table S8……12
Fig S1……13
Fig S2……14
Fig S3……15
Fig S4……16
Fig S5……17
Fig S6……18
Fig S7……18
Methods……20
Table S8……17
References……19

Table S1. Physicochemical characteristics of the 30 studied rivers: pH, temperature (Temp, ^{o}C), altitude (alt, m), latitude (Lat, degree), longitude (Long, degree), channel width (Width, m), water depth (cm), days of exposure (days), nitrate (N, mg/ L), orthophosphate (P, mg/ L), dissolved organic carbon (DOC, mg/L), canopy cover (Canopy), quantity of cobbles, quantity of gravel, amount of sand, quantity of silt, quantity of leaf litter in the open channel, quantity of submerged plants, quantity of submerged wood. Canopy cover, sediment morphology (cobbles, gravel, sand and silt) and the quantity of leaf litter, submerged plants (Sub plant) and submerged wood (Sub wood) were characterized semi–quantitatively in situ at each site (giving values ranging from 0 when no presence to a maximum of 3). Physicochemical measurements of pH, altitude, latitude, longitude, dissolved organic carbon, dissolved inorganic nitrogen, ammonium, nitrate and phosphate were obtained from the UK Environment Agency as annual averages when available.

River	pH	Temp	Alt	Lat	Long	Width	Water depth	Days of exposure	N	P	DO	Amo	DIN	Canopy	Cobbles	Gravel	Sand	Silt	Leaf litter	Sub plant	Sub wood
Anton	8.81	7.75	44.00	51.15	1.46	15.00	44.33	45.00	8.11	0.03	11.43	0.03	8.14	0.00	0.00	2.67	2.00	0.00	0.00	0.67	0.33
B.brooks	7.65	11.97	11.00	51.44	0.25	5.50	16.00	30.00	18.60	0.34	7.32	0.19	18.79	0.00	0.33	2.33	1.67	0.67	1.67	0.00	1.33
Broadstone	5.50	7.13	71.00	51.09	0.06	1.50	9.67	29.00	3.33	0.31	9.61	0.04	3.37	3.00	1.33	2.67	0.00	0.00	1.67	0.00	1.00
Bure	8.12	8.67	20.00	52.82	1.20	8.50	47.33	31.00	6.23	0.06	10.32	0.04	6.27	1.00	0.00	2.00	3.00	0.00	0.00	0.00	0.00
Crowdudle	7.98	6.76	103.00	54.65	-2.60	12.00	18.00	45.00	0.89	0.01	11.43	0.01	0.90	3.00	1.67	2.00	1.33	0.00	1.33	0.00	0.33
Deathwater	7.68	6.96	76.00	51.11	0.85	3.00	45.00	29.00	0.88	0.03	9.08	0.03	0.91	3.00	0.00	0.33	3.00	0.67	2.00	0.00	0.67
Gl1	5.90	7.09	190.99	52.10	-3.84	5.50	16.33	43.00	0.23	0.01	9.00	0.03	0.26	3.00	2.33	1.67	0.00	0.00	0.67	0.00	1.00
Glaven	8.11	8.04	14.00	52.90	1.06	4.50	51.33	31.00	6.75	0.29	10.61	0.06	6.81	3.00	0.00	0.00	2.67	1.00	1.00	0.00	0.33
Howe.beck	8.00	7.41	168.99	54.61	-2.59	5.00	25.00	49.00	0.67	0.01	13.35	0.01	0.68	0.00	3.00	1.00	0.00	0.00	0.00	0.00	0.00
Kennet	7.72	8.21	124.99	51.42	1.72	12.00	19.67	45.00	7.51	0.03	10.05	0.03	7.54	1.00	0.00	3.00	1.00	0.00	0.33	0.00	0.00
Lamports	7.31	9.53	99.99	51.15	1.72	3.00	6.83	29.00	4.58	0.05	9.71	0.04	4.62	2.00	1.00	3.00	0.33	0.00	0.00	0.33	0.00
Leith	8.33	6.67	108.99	54.61	-2.62	6.50	26.00	49.00	2.21	0.05	9.00	0.13	2.34	0.00	2.33	1.67	0.00	0.00	1.00	0.67	0.00
Ll3	6.12	6.80	354.98	52.14	-3.73	1.50	13.67	43.00	0.48	0.00	9.00	0.13	0.61	0.00	3.00	1.00	0.00	0.00	1.00	0.00	0.00
Ll6	6.67	7.29	294.99	52.13	-3.72	1.50	18.33	43.00	0.04	0.00	9.00	0.13	0.17	1.00	2.67	1.33	0.00	0.00	0.67	0.00	0.00
Ll7	6.89	7.09	340.98	52.13	-3.75	1.50	14.33	43.00	0.03	0.01	9.00	0.13	0.16	1.00	2.33	1.67	0.00	0.00	0.33	0.67	0.33
Ll8	5.23	7.56	316.99	52.16	-3.75	1.50	19.67	43.00	0.30	0.01	9.00	0.13	0.43	0.00	2.33	1.67	0.00	0.00	0.33	0.00	0.00
Loan.Oak	6.40	6.92	118.99	51.08	0.10	2.00	8.33	29.00	0.55	0.03	9.77	0.03	0.58	3.00	3.00	1.00	0.00	0.00	1.67	0.00	0.33
Loddon	7.61	9.55	60.00	51.42	1.72	5.00	54.67	45.00	12.04	0.17	8.51	0.03	12.07	0.00	0.00	3.00	0.67	0.00	1.00	0.67	0.33

Continue Table S1

Lyde	8.19	9.55	60.00	51.29	1.72	4.00	27.33	45.00	7.03	0.01	10.17	0.03	7.06	2.00	0.00	3.00	1.00	0.00	0.67	0.00	0.00
Lyvennet	8.57	6.76	109.99	54.61	-2.62	8.00	47.33	49.00	1.11	0.02	11.60	0.03	1.14	0.00	3.00	1.00	0.00	0.00	1.00	0.00	0.00
Morland.beck	8.40	6.71	115.00	54.61	-2.61	4.50	32.00	49.00	1.54	0.02	11.95	0.03	1.57	2.00	2.33	1.67	0.00	0.00	1.00	0.00	0.00
Nadder	7.76	8.62	111.99	51.23	1.72	0.80	15.67	29.00	9.30	0.27	8.54	0.98	10.28	1.00	0.00	3.00	1.00	1.00	1.00	0.00	0.00
Oak.hanger	7.58	9.29	79.00	51.12	0.90	2.00	41.67	29.00	2.09	0.18	9.51	0.04	2.13	2.00	0.00	0.00	0.00	3.00	3.00	0.00	2.00
Old.lodge	6.60	6.76	109.66	51.05	0.08	1.50	7.00	29.00	1.06	0.08	9.68	0.03	0.58	3.00	3.00	1.33	0.00	0.00	2.67	0.00	1.33
Stiffkey	7.80	8.05	10.00	52.92	0.89	4.50	47.33	31.00	7.00	0.13	10.61	0.03	7.03	1.00	0.00	2.33	2.00	0.33	0.33	1.00	0.00
Tatt	7.42	7.27	47.00	52.82	0.75	3.00	37.33	33.00	8.29	0.07	9.49	0.03	8.32	3.00	0.00	0.67	1.33	2.00	2.00	0.67	1.00
Test	8.08	8.04	43.00	51.14	1.47	15.00	51.33	45.00	8.17	0.05	10.68	0.03	8.20	0.00	0.00	1.67	2.33	0.67	1.67	1.67	0.00
Waveney	7.54	6.90	10.00	52.42	1.36	9.00	41.33	61.00	6.11	0.09	10.17	0.06	6.17	2.00	0.00	1.33	2.67	0.33	0.67	1.00	0.00
Wennsum	7.24	7.27	10.00	52.78	0.95	5.00	39.33	61.00	6.11	0.09	10.17	0.04	6.16	0.00	0.00	3.00	1.00	0.00	0.00	1.00	0.33
Wey	7.77	7.61	44.00	51.19	0.68	21.00	21.33	29.00	4.61	0.15	9.63	0.04	4.65	2.00	0.67	2.00	2.33	0.00	1.00	0.00	0.00

Table S2. Identified Taxa list in the benthic (B) and hyporheic (H) zones for the 30 studied streams during the study period from October 2016 to December 2016: Anton (Ant), Beverly Brook (B.br), Broadstone (Bro), Bure (Bur), Crowdundle (Cro), Deathwater (Dea), GI1, Glaven (Gla), Howe Beck (How), Kennet (Ken), Lamports (Lam), Leith (Lei), LI3, LI6, LI7, LI8, Lone oak (Loa), Loddon (Lod), Lyde (Lyd), Lyvennet (Lyv), Morland Beck (Mor), Nadder (Nad), Oak.hanger (Oak), Old lodge (Old), Stiffkey (Sti), Tatt (Tat), Test (Tes), Waveney (Wav), Wennsum (Wen), Wey. Taxonomic resolution = species (.sp), tribu (Tr.), sub-family (Sf.), family (F.), order (O.), sub-class (Sc.). Abbreviations for taxa names used in Fig. S5 are given in brackets.

Taxa	Zone	Stream
EUMETAZOA INVERTEBRATES		
Non-insect		
Nematoda (Nema)	B/H	Ant, B.br, Boa, Cro, Dea, GI1, Gla, How, Ken, Lam, Lei, LI3, LI6, LI7, Lod, Lyd, Lyv, Mor, Nad, Oak, Sti, Tat, Tes, Wav, Wen, Wey
Acari (Aca)	B	Ant, Det, Lam, Lyv, Wen
Asellus sp. (Ase)	B	B.br, Dea, Gla, Lam, Lei, Tes, Wav
Gammarus sp. (Gamma)	B/H	Ant, B.br, Bur, Cro, Dea, Gla, How, Ken, Lam, Lei, LI7, Lod, Lyd, Lyv, Mor, Nad, Sti, Tat, Tes, Wav, Wen, Wey
Gastotricha (Gastro)	B	B.br
Sc. Oligochaeta (Oli)	B/H	Ant, B.br, Bro, Bur, Crow, Dea, GI1, Gla, How, Ken, Lam, Lei, LI3, LI6, LI7, LI8, Loa, Lod, Lyd, Lyv, Mor, Nad, Oak, Old, Sti, Tat, Tes, Wav, Wen, Wey
Cl. Ostracoda (Ostra)	B/H	Ant, Bro, Lam, LI8, Lod, Lyd, Old, Tat, Tes, Wen
Sphaerium sp (Sphae)	B	Lod
Corophium sp. (Coro)	B	Lei, Wen
Sc. Hirudinea (Hiru)	B/H	Ant, B.br, Cro, Gla, How, Ken, Lei, Lod, Mor, Sti, Tes
O. Harpacticoida (Harpa)	B/H	Ant, B.br, Gla, Ken, Lam, Lei, Loa, Lyd, Lyv, Mor
Insects (O. Odonata)		
Calopterix sp. (Calo)	B	B.br
Insects (O. Plecoptera)		
Amphinemura sp. (Amphi)	B	Cro, LI7
Isoperla sp. (Iso)	B/H	Ant, Cro, Dea, GI1, How, Ken, LI·, LI6, LI7, LI8, Loa, Lyv, Mor, Old, Sti
Leuctra sp. (Leu)	B	Cro, GI1, LI3, LI7, LI8, Loa, Lyv
Insects (O. Ephemeroptera)		
Baetis sp. (Bae)	B	Ant, Bur, How, Ken, Lei, Lyd
Caenis sp. (Cae)	B	Gla, Kenn, Lyv, Wav, Wey
Ephemerella sp. (Ephe)	B	Ken, Lam
Paraleptophlebia sp. (Para)	B	How, Mor
Insects (O. Trichoptera)		
F. Leptoceridae (Lepto)	B	Ant

Continue Table S2

Hydropsyche sp. (Hydro)	B	Ant, GI1, Lud, Lyv, Mor, Wav, Wen, Wey
Hyporyacophila sp. (Hypo)	B	Lam
Ithytrichia sp. (Ithy)	B	Ant, Bur, Lyd, Lyv, Sti, Tes, Wen
Lepidostoma sp. (Lepi)	B	Gla, Sti, Wen
F. Leptoceridae (Lepto)	B	Lam
Plectrocnemia sp. (Plectro)	B	LI3
Polycentropus sp. (Poly)	B	Sti
Prosorhyacophila sp. (Proso)	B	Lyd
Rhyacophila sp. (Rhya)	B	GI1, How, Ken, Mor
Nemotaulius sp. (Nemo)	B	Tes
Sericostoma sp. (Seri)	B	Ant, GI1, How, Lyv, Tes, Wav, Wen
Sf. Agapetinae (Aga)	B	Lyd
Sf. Pseudoneuroclipsis (Pseu)	B	Lyd
Wormaldia sp. (Worma)	B	Ken
Silo sp. (Silo)	B	Ant
Oecetis sp. (Oe)	B	Tes
Insects (O. Diptera)		
Chironomidae pupae (NA)	B	Dea, Ken, Nad, Wav, Wen
Sf. Ceratopogoninae (Cera)	B	Ant, Bro, Gla, How, Lam, Lei, LI3, LI8, Lyv, Stif, Tat, Tes, Wey
Sf. Clinocerinae (Clino)	B	Ant, GI1, Lei, Lyv
Sf. Hemerodromiinae (Heme)	B	Bur, GI1, Lam, Lyd
Sf. Orthocladinae (Ortho)	B/H	Ant, B.br, Bro, Bur, Bur, Cro, Dea, GI1, Gla, How, Ken, Lam, Lei, LI·, LI6, LI7, LI8, Loa, Lod, Lyd, Lyv, Mor, Nad, Old, Sti, Tat, Tes, Wav, Wen, Wey
Sf. Tanypodinae (Tany)	B/H	Ant, B.br, Bro, Cro, Dea, GI1, Gla, How, Ken, Lam, Lei, LI3, Loa, Lod, Lyd, Lyv, Mor, Oak, Old, Sti, Tes, Wav, Wen
F. Stratyomiidae (Straty)	B	Cro, Lyv
Tr. Chironomini (Chiro)	B/H	Ant, B.br, Bor, Bur, Cro, Dea, GI1, Gla, How, Ken, Lei, LI3, LI6, Loa, Lod, Lyd, Lyv, Mor, Nad, Oak, Sti, Tat, Nad, Wav, Wey
Tr. Limoniini (Limo)	B	Cro, LI6, Mor, Tat, Tes, Wav
Tr. Pediciini (Pedi)	B/H	Gla, LI7, Lyd, Wey
Tr. Simuliini (Simu)	B	GI1, Gla, How, Lei, Old, Wav, Wen
Tr. Tanytarsini (Tany)	B/H	Ant, B.br, Bro, Bur, Cro, GI1, How, Ken, Lam, Lei, Lod, Lyd, Lyv, Mor, Nad, Old, Tat, Tes, Wav, Wen, Wey
F. Anthomyiidae (Antho)	B	Lam
Tr. Hexatomini (Hexa)	B	LI7
Insects (O. Coleoptera)		
Elmis sp. Adult (Elmis (A))	B	Ant, Gla, Lyd
Elmis sp. Larva (Elmis (L))	B/H	Ant, Gla, Ken, Lam, Lei, Lod, Mor, Sti, Tes, Wen
Haliplus sp. (Hali)	B	How, Ken, Lei, Lyv, Mor, Wav, Wey
Limnius sp. Adult (Limni (A))	B	How, Sti
Limnius sp. Larva (Limni (L))	B/H	Ant, Cro, GI1, Gla, Lei, Sti, Tes, Wav, Wen
Orectochilus sp. Larva (Ore (L))	B	Lyv
Riolus sp larva (Rio (L))	B	Lyv

Continue Table S2

CILIATES

Sc. Cyrtophorida (Cyrto)	B/H	Ant, B.br, Bro, Bur, Cro, Dea, How, Ken, Lam, LI6, LI8, Loa, Lod, Wen
Sc. Gymnostomatida (Gymno)	B/H	Ant, B.br, Bro, Bur, Cro, Dea, GI1, Gla, How, Ken, Lam, Lei, LI3, Li6, Li7, LI8, Loa, Lod, Lyd, Lyv, Mor, Nad, Oak, Old, Tes, Wav, Wen, Wey
Sc. Heterotrichia (Hete)	B/H	Ant, B.br, Bro, Bur, Cro, Dea, GI1, Gla, How, Ken, Lam, Lei, LI3,LI6, LI7, Loa, Lod, Lyv, Mor, Nad, Oak, Old, Sti, Tat, Wav, Wen, Wey
Sc. Hymenostomata (Hyme)	B/H	Ant, B.br, Bro, Bur, Cro, Dea, GI1, Gla, How, Ken, Ken, Lam, Lei, LI3, LI6, LI7, LI8, Loa, Lod, Lyd, Luv, Mor, Nad, Oak, Old, Sti, Tat, Tes, Wav, Wen, Wey
Sc. Hypotrichia (Hypo)	B/H	Ant, B.br, Bro, Bur, Cro, Dea, GI1, Gla, How, Ken, Lam, Lei, LI3, LI6, LI7, LI8, Loa, Lod, Lyd, Lyv, Mor, Nad, Oak,Old, Stif, Tes, Wav, Wen, Wey
Sc. Odontostomatida (Odo)	B/H	B.br, Dea, Gla, Lod, Lyv, Old, Tat
Sc. Oligotrichia (Oli)	B/H	Ant, Bro, Cro, LI3, LI6, LI7, Loa, Sti, Wen
Sc. Peritrichia (Peri)	B/H	Bro, Bur, Cro, Dea, GI1, Gla, How, Ken, Lam, Lei, LI6, LI7, LI8, Loa, Lod, Lyd, Lyv, Mor, Nad, Oak, Old, Sti, Tat, Tes, Wen, Wey
Sc. Pleurostomatida (Pleuro)	B/H	Ant, B.br, Bro, Bur, Cro, Dea, GI1, How, Ken, Lam , Lei, LI3, LI6, LI7, LI8, Loa, Lyd, Mor, Old, Stif, Tat, tes, Wav, Wen, Wey
Sc. Prostomatida (Prosto)	B/H	Ant, B.br, Bro, Bur, Cro, Dea, GI1, Gla, How, Ken, Lam, LI8, Loa, Lod, Mor, Nad, Old, Stif, Tes, Wen, Wey
Sc. Suctoria (Sucto)	B/H	Ant, B.br, Bro, Bur,C ro, Dea, Gla, How, Ken, Lam, Lei, LI3, LI6, LI7, LI8, Loa, Lod, Lyd, Lyv, Mor, Nad, Oak, Old, Tat, Tes, Wav, Wen, Wey
Cyst (Cyst)	B/H	Ant, B.br, Bro, Bur, Cro, Dea, Gla, How, Ken, Lam, Lei, LI8, Loa, Lod, Lyd, Lyv, Mor, Nad, Oak, Old, Tat, Wav, Wen, Wey

FLAGELATES

Flagelates (Fla)	B/H	Ant, B.br, Bro, Bur, Cro, Dea, GI1, Gla, How, Lod, Lyd, Luv, Mor, Nad, Oak, Old, Sti, Tat, Tes, Wav, Wen, Wey

Table S3. Fitting coefficients of streambed compartment, decay rates, biological vectors and environmental vectors against the NDMS ordination model. Note that only significant values are reported. NO_3 = nitrate; DIN = dissolved inorganic nitrogen; Inv. biomass = invertebrate biomass; Prot. biomass = protozoa biomass; Inv. diversity = Eumetazoa invertebrate diversity; Prot. diversity = Protozoa diversity; Prok. met. rich = Prokaryota metabolic richness; Prok. met. div. = Prokaryota metabolic diversity; k_{cotton} = decay coefficient cotton strips; k_{green} = decay coefficient green–tea; S = long–term carbon stabilization factor

**Fitted variables**	**NMDS1**	**NMDS2**	**R^2**	**P**	**sig**
**Factor (Zone)**	--	--	0.553	0.001	***
BZ	-0.474	-0.034			
HZ	0.474	0.034			
**Vectors**					
Inv. diversity	-0.945	0.328	0.773	0.001	***
k_{cotton}	-0.888	0.459	0.651	0.001	***
k_{green}	-0.875	-0.484	0.282	0.001	***
Prot. diversity	-0.935	-0.354	0.388	0.001	***
S	0.999	0.045	0.485	0.001	***
Body size	-0.889	0.459	0.169	0.003	**
Prok. met. rich	-0.684	-0.730	0.175	0.003	**
Inv. biomass	-0.884	0.467	0.175	0.004	**
pH	-0.445	0.896	0.169	0.004	**
DIN	-0.158	0.987	0.152	0.009	**
NO_3	-0.164	0.987	0.154	0.009	**
Prok. met. div.	-0.484	-0.875	0.129	0.016	*
Altitude	0.146	-0.989	0.118	0.038	*

Table S4. ANOVA tables for the comparison of ecological variables (taxonomic group biomass, taxonomic α-diversity, bacterial functional diversity, EcoPlate AWCD and substrates AWCD) between benthic zone and hyporheic zone. Significance codes: 0 (***), 0.001 (**), 0.01 (*) and 0.05 (·). A Kenward-Roger approximation was used to calculate the effective degrees of freedom (DF).

	Sum Sq	DF	Den DF	F value	P (>F)	Sig
**(1) Log_{10} eumetazoa invertebrates biomass**						
Zone	191.49	1	130.56	202.55	< 0.001	***
**(2) Log_{10} protozoa biomass**						
Zone	93.50	1	131.26	168,09	< 0.001	***
Log_{10} bacterial biomass ~ zone						
Zone	1,56	1	130,79	2.79	0,096	.
**(3) α-diversity eumetazoa invertebrates**						
Zone	2687.10	1	129.22	257.13	< 0.001	***
**(4) α-diversity protozoa**						
Zone	640.12	1	131.17	131.07	< 0.001	***
**(5) Bacterial functional diversity (S-W, EcoPlate)**						
Zone	5.63	1	134.24	9.87	0,002	**
**(6) EcoPlate AWCD**						
Zone	0.32	1	134.04	19.57	< 0.001	***
**(7) Amino acids AWCD**						
Zone	0.15	1	134.26	4.34	0,039	*
**(8) Carbohydrates AWCD**						
Zone	0.50	1	134.13	22.46	< 0.001	***
**(9) Carboxylic acids AWCD**						
Zone	0.23	1	134.78	11.51	0,001	***
**(10) Phenolic compunds AWCD**						
Zone	0.17	1	134.99	5.12	0,025	*
**(11) Amines AWCD**						
Zone	0.12	1	134.70	4.46	0,036	*
**(12) Polymers AWCD**						
Zone	0.79	1	133.75	25.92	< 0.001	***

Table S5. Summary table of the fitted predictive equations after the casual three steps method routine for decay rate of cotton–strips (k_{cotton}). Details: Marginal R^2 of the model (Mar R^2), conditional R^2 of the model (Cond R^2), proportion of mediated effect (Prop. Mediated), Partial R^2, Standardised coefficients (Std β), standard errors (SE), degrees of freedom (DF), t values, lower (Low) and upper (Up) limits of the credible intervals (CrI) and P values (P). Significance codes (Sig): 0 (***), 0.001 (**), 0.01 (*) and 0.05 (·). A Satterthwaite approximation was used to calculate the effective degrees of freedom.

	Model	Partial R^2	Std β	SE	DF	t value	Low 95%CrI	Up 95%CrI	P	Sig
Step 1	**k_{cotton} ~ Zone**<br>Marg R^2= 0.3094<br>Cond R^2 = 0.5188									
	**Intercept**	--	0.0061	0.0006	13.8200	10.2950	0.0050	0.0073	--	--
	**Zone**	0.3094	**-0.0041**	0.0004	128.4000	-9.9380	-0.0050	-0.0033	>0.001	***
Step 2	**Inv. Biomass ~ Zone**<br>Marg R^2= 0.3910<br>Cond R^2 = 0.7080									
	**Intercept**	--	0.6234	0.1763	11.6302	3.5130	0.2875	0.9564	--	--
	**Zone**	0.3910	-1.2618	0.0888	129.7889	-14.2560	-1.4390	-1.0900	>0.001	***
	**Inv. div. ~ Zone**<br>Marg R^2= 0.4011<br>Cond R^2 = 0.7651									
	**Intercept**		0.6677	0.1693	11.3062	3.9200	0.3223	0.9966	--	--
	**Zone**	0.4011	0.6677	0.0796	128.6020	-16.0500	-1.4379	-1.1283	>0.001	***
Step 3	**k_{cotton} ~ Zone × Inv. biomass + Zone × Inv. div.**<br>Marg R^2= 0.5668<br>Cond R^2 = 0.6308<br>Prop. Mediated = **68%**									
	**Intercept**	--	0.0037	0.0005	18.6300	8.0310	0.0027	0.0045	--	--
	**Zone**	0.0330	**-0.0013**	0.0006	94.9700	-2.1130	-0.0024	-0.0001	0.0372	**
	**Inv. Biomass**	0.1120	0.0022	0.0005	143.7000	4.3420	0.0012	0.0031	>0.001	***
	**Inv. Div.**	0.0830	0.0016	0.0005	102.9000	3.6220	0.0008	0.0026	>0.001	***
	**Zone × Inv. Biomass**	0.0410	-0.0015	0.0006	140.9000	-2.6290	-0.0026	-0.0004	>0.001	***
	**Zone × Inv. div.**	0.0350	-0.0014	0.0006	104.0000	-2.3610	-0.0027	-0.0003	0.0201	**

Inv.biomass: Log_{10} biomass of Eumetazoa invertebrates, Inv. Div.: α–diversity of Eumetazoa invertebrates, Zone: Streambed compartment (2 levels factor: HZ and BZ).

Table S6. Summary table of the fitted predictive equations after the casual three steps method routine for decay rate of green–tea (k_{green}). See Table S5 for details.

	Model	Partial R^2	Std β	SE	DF	t value	Low 95%CrI	Up 95%CrI	P	sig
**Step 1**	**k_{green} ~ Zone**<br>Marg R^2= 0.2800<br>Cond R^2 = 0.6450									
	**Intercept**	--	0.0031	0.0030	9.7040	23.2300	0.0028	0.0033	--	--
	**Zone**	0.2800	**-0.0008**	0.0001	129.2000	-10.9600	-0.0009	-0.0006	>0.001	***
**Step 2**	**Prot. biomass ~ Zone**<br>Marg R^2= 0.4319<br>Cond R^2 = 0.6098									
	**Intercept**	--	0.6229	0.1182	10.5666	5.2490	0.3922	0.8641	--	--
	**Zone**	0.4319	-1.3379	0.1029	130.0019	-12.9930	-1.5428	-1.1244	>0.001	***
	**Inv. div. ~ Zone**<br>Marg R^2= 0.4011<br>Cond R^2 = 0.7651									
	**Intercept**	--	0.6680	0.1690	11.3060	3.9200	0.3220	0.9970	--	--
	**Zone**	0.4011	0.6680	0.0800	128.6020	-16.0500	-1.4380	-1.1280	>0.001	***
**Step 3**	**k_{green} ~ Zone + Prot. biomass + Zone × Inv. div.**<br>Marg R^2= 0.3995<br>Cond R^2 = 0.7083<br>Prop. Mediated = **62%**									
	**Intercept**	--	0.0030	0.0001	15.7500	23.1600	0.0028	0.0001	--	--
	**Zone**	0.0390	**-0.0003**	0.0001	149.2000	-2.9420	-0.0006	-0.0001	0.0038	**
	**Prot. Biomass**	0.1040	0.0002	0.00005	148.7000	4.9210	0.0001	0.0003	0.0000	***
	**Inv. Div.**	0.0100	-0.0001	0.0001	150.2000	-1.2960	-0.0002	0.0001	0.1969	
	**Zone × Inv. Div.**	0.0450	0.0003	0.0001	146.0000	3.3030	0.0001	0.0005	0.0012	**

Prot.biomass: Log_{10} biomass of Protozoa, Inv. Div.: α–diversity of Eumetazoa invertebrates, Zone: Streambed compartment (2 levels factor: HZ and BZ).

Table S7. Summary table of the fitted predictive equations after the casual three steps method routine for decay rate of red–tea (k_{red}). See Table S5 for details.

	Model	Partial R^2	Std β	SE	DF	t value	Low 95%CrI	Up 95%CrI	P	sig
**Step 1**	**k_{red} ~ Zone**<br>Marg R^2= 0.0883<br>Cond R^2 = 0.5571									
	**Intercept**	--	0.00082	0.00004	10.33000	22.05000	0.00075	0.00089	--	--
	**Zone**	0.08835	**-0.00011**	0.00002	123.10000	-5.37000	-0.00015	-0.00007	0.00000	***
**Step 2**	**Prot. biomass ~ Zone**<br>Marg R^2= 0.43190<br>Cond R^2 = 0.60980									
	**Intercept**	--	0.62290	0.11820	10.56660	5.24900	0.39220	0.86410	--	--
	**Zone**	0.43190	-1.33790	0.10290	130.00190	-12.99300	-1.54280	-1.12440	>0.001	***
	**Prok. biomass ~ Zone**<br>Marg R^2= 0.01160<br>Cond R^2 = 0.37280									
	**Intercept**	--	0.11829	0.15460	11.55320	0.76900	-0.19909	0.42959	--	--
	**Zone**	0.40110	-0.18583	0.13180	127.92410	-1.41800	-0.45449	0.07401	0.09630	·
**Step 3**	**k_{green} ~ Zone + Prot.biomass + Prok.biomass.**<br>Marg R^2= 0.39950<br>Cond R^2 = 0.70830<br>Prop. Mediated = **81%**									
	**Intercept**	--	0.00078	0.00004	11.94000	20.68200	0.00003	0.00085	--	--
	**Zone**	0.00300	**-0.00002**	0.00003	138.40000	-0.89600	-0.00008	0.00003	0.37182	
	**Prot. biomass**	0.06600	0.00006	0.00002	145.00000	3.85300	0.00003	0.00009	0.00017	***
	**Prok. biomass**	0.03600	0.00003	0.00001	145.10000	2.70900	0.00001	0.00006	0.00755	**

Prot.biomass: Log_{10} biomass of Protozoa, Prok.biomass: Log_{10} biomass of Prokariota, Zone: Streambed compartment (2 levels factor: HZ and BZ).

Table S8. Summary table of the fitted predictive equations after the casual three steps method routine for stabilization factor coefficient (S[illegible]). See Table S5 for details.

Step	Model	Partial R^2	Std β	SE	DF	t value	Low 95%CrI	Up 95%CrI	P	sig
Step 1	**S ~ Zone**<br>Marg R^2= 0.2800<br>Cond R^2 = 0.6450									
	**Intercept**	--	0.2837	0.0221	12.2206	12.8700	0.2398	0.3257	--	--
	**Zone**	0.2853	**0.1261**	0.0116	126.6092	10.8600	0.1028	0.1491	>0.001	***
Step 2	**Inv. Biomass ~ Zone**<br>Marg R^2= 0.3910<br>Cond R^2 = 0.7080									
	**Intercept**	--	0.6234	0.1763	11.6302	3.5130	0.2875	0.9564	--	--
	**Zone**	0.3910	-1.2618	0.0888	129.7889	-14.2560	-1.4390	-1.0900	>0.001	***
	**Prot. Biomass ~ Zone**<br>Marg R^2= 0.6098<br>Cond R^2 = 0.4319									
	**Intercept**	--	0.6229	0.1182	10.5666	5.2490	0.3922	0.8641	--	--
	**Zone**	0.4319	-1.3379	0.1029	130.0019	-12.9930	-1.5428	-1.1244	>0.001	***
	**Prok. F. Div. ~ Zone**<br>Marg R^2= 0.4320<br>Cond R^2 = 0.6098									
	**Intercept**	--	0.6185	0.1182	10.5666	5.2490	0.3905	0.8545	--	--
	**Zone**	0.4320	-1.3369	0.1029	130.0019	-12.9930	-1.5380	-1.1436	>0.001	***
Step 3	**S ~ Zone + Inv. Biomass + Prot. Biomass + Prok. f. div.**<br>Marg R^2= 0.3995<br>Cond R^2 = 0.7083<br>Prop. Mediated = **59%**									
	**Intercept**	--	0.3185	0.0211	13.8781	15.1720	0.2776	0.3607	--	--
	**Zone**	0.0340	**0.0513**	0.0195	145.0262	2.6510	0.0129	0.0924	0.0089	**
	**Inv. Biomass**	0.0310	-0.0201	0.0101	147.3874	-2.0380	-0.0396	-0.0007	0.0434	*
	**Prot. Biomass**	0.0680	-0.0323	0.0085	139.7118	-3.7680	-0.0482	-0.0167	0.0002	***
	**Bac. F. Div.**	0.0100	-0.0124	0.0062	136.1964	-1.9780	-0.0235	-0.0002	0.0500	*

Inv.biomass: Log_{10} biomass of Eumetazoa invertebrates, Prot.biomass: Log_{10} biomass of Protozoa, Prok.f.div: Prokariotic funcional diversity, Zone: Streambed compartment (2 levels factor: HZ and BZ).

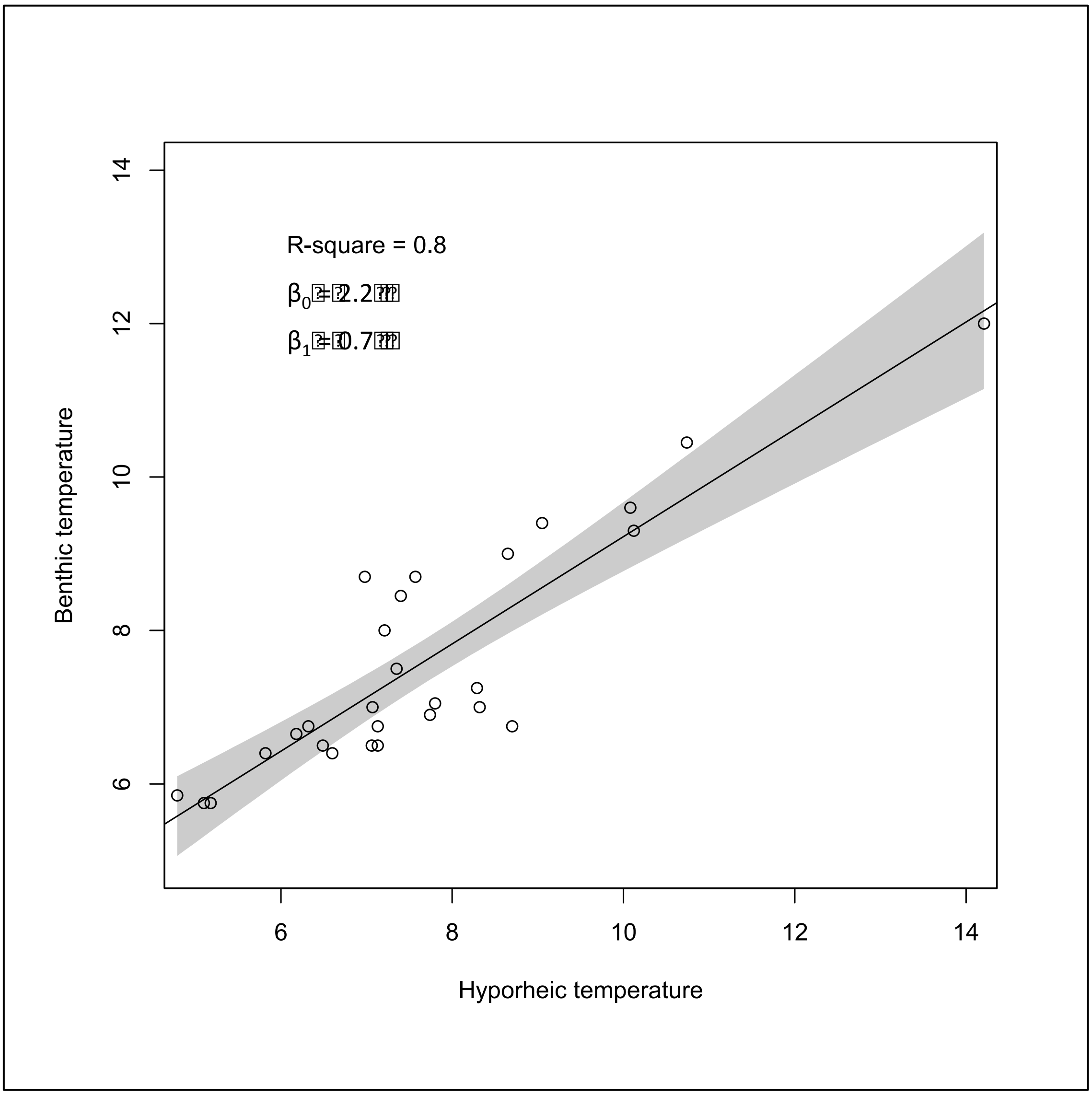

Fig S1. Linear regression model predicting benthic temperature (ºC) as a response of the hyporheic temperature (covariate). β_0: intercept, β_1: slope, R-square: coefficient of determination.

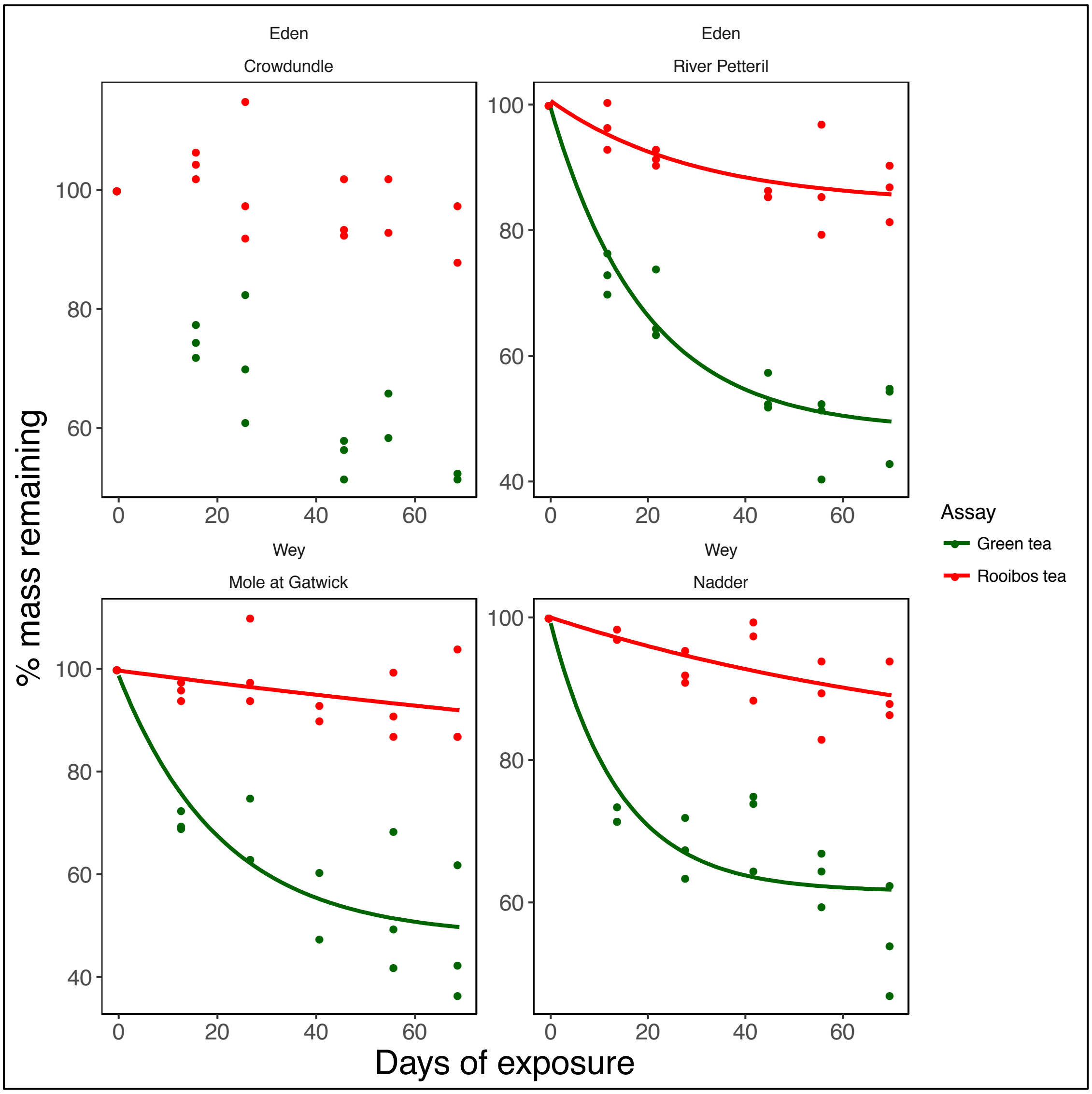

Fig S2: Decay coefficients of green and rooibos–tea bioassays from sites in two of the study catchments. The negative exponential dacay model is fitted to the data (where possible) highlighting that the k used in the analysis is generally supported for these assays. Data were collected by volunteers from the Eden Trust and Surrey Wildlife Trust as part of a Citizen Science Project: http://www.riverflies.org/scratching-below-surface-monitoring-functioning-under-

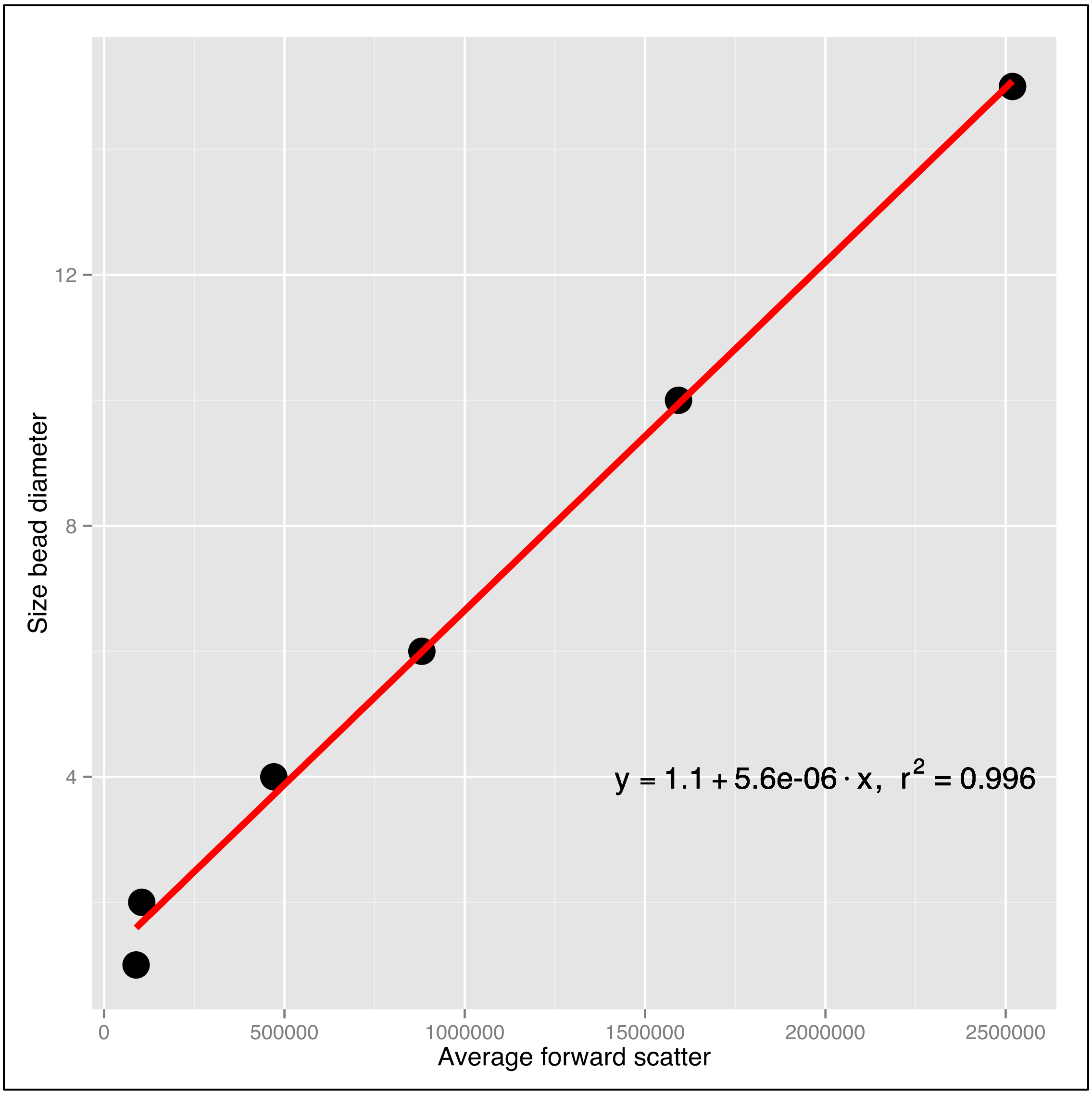

Fig S3. Cell size calibration from flow cytometer data. Cell size (μm) was estimated from the relationship between calibration beads of known size and forward scatter values returned by flow cytometer.

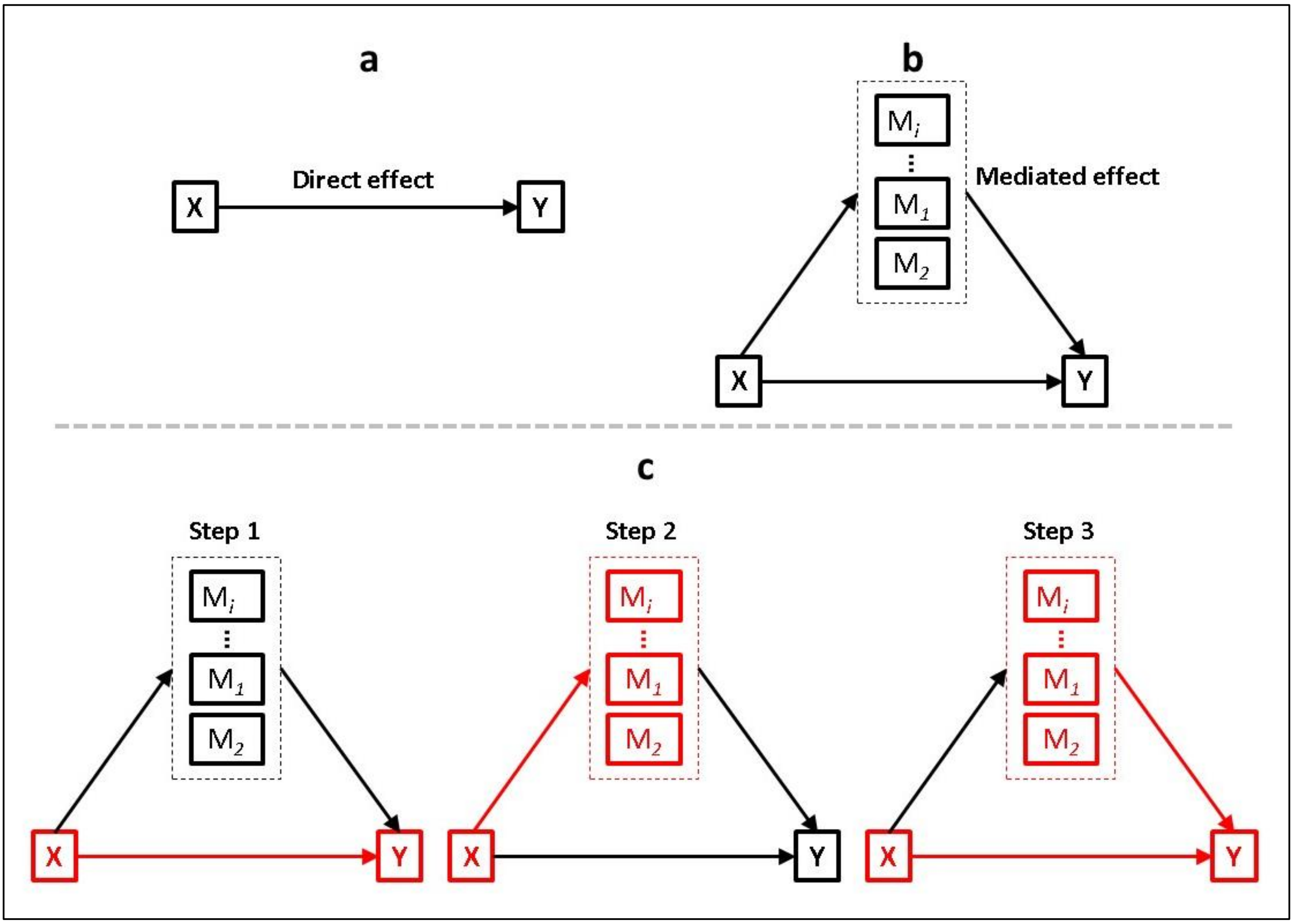

Fig S4. (a) Path diagram of a direct effect model. The variation in the response (Y) is explained just by the exposure variable (X) and the associate error. (b) Path diagram of a multiple mediation model. The variation in Y is explained by direct effect of X and indirect effect mediated by the mediators ($M_{1,2\ldots i}$). (c) Diagram of the casual three–steps method proposed by Judd and Kenny (1981).

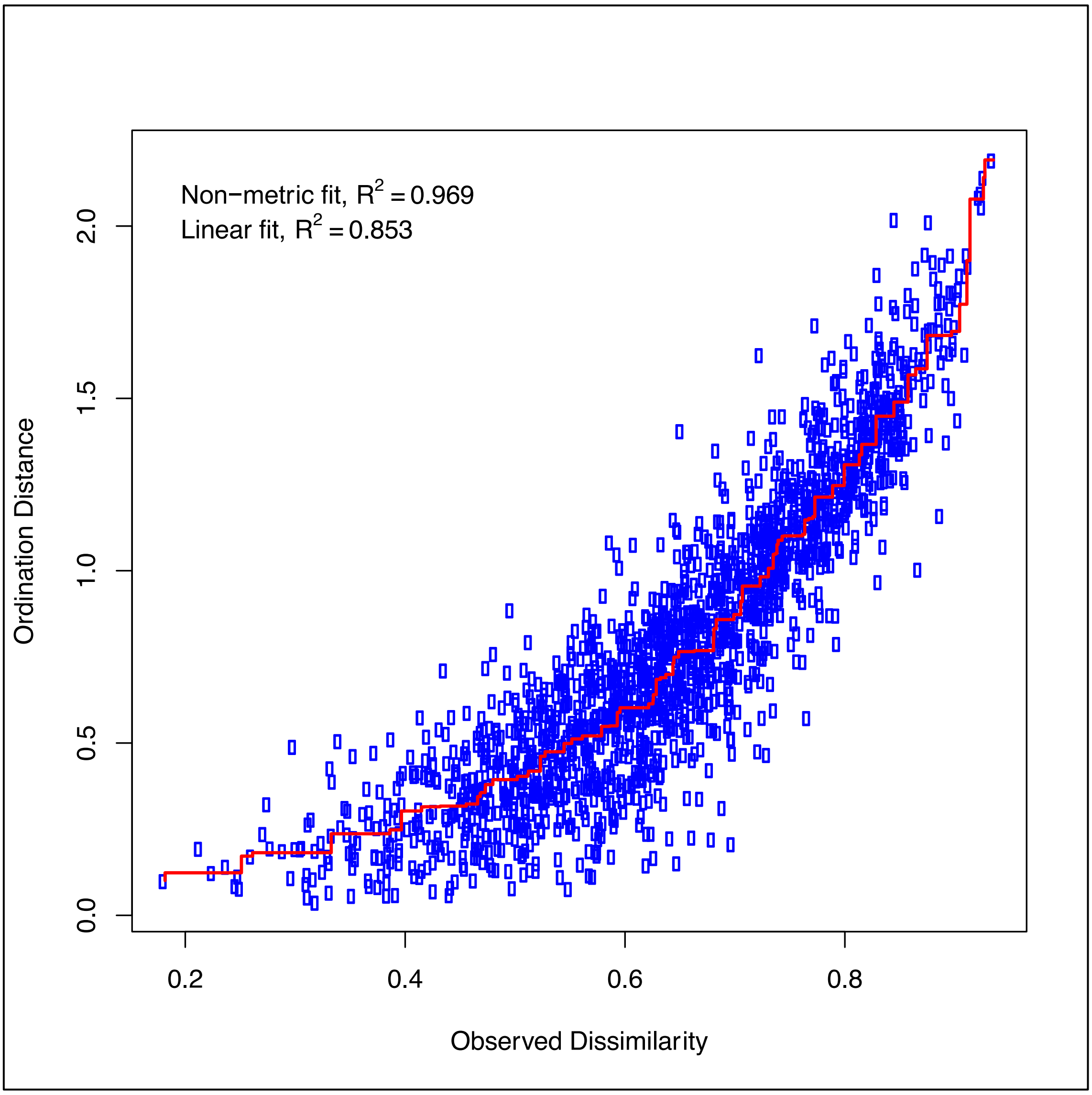

Fig S5. Shepard–plot of the NDMS ordination. Scatter around the regression of distances between each pair of communities against their original dissimilarities. The fit is shown as a monotone step line.

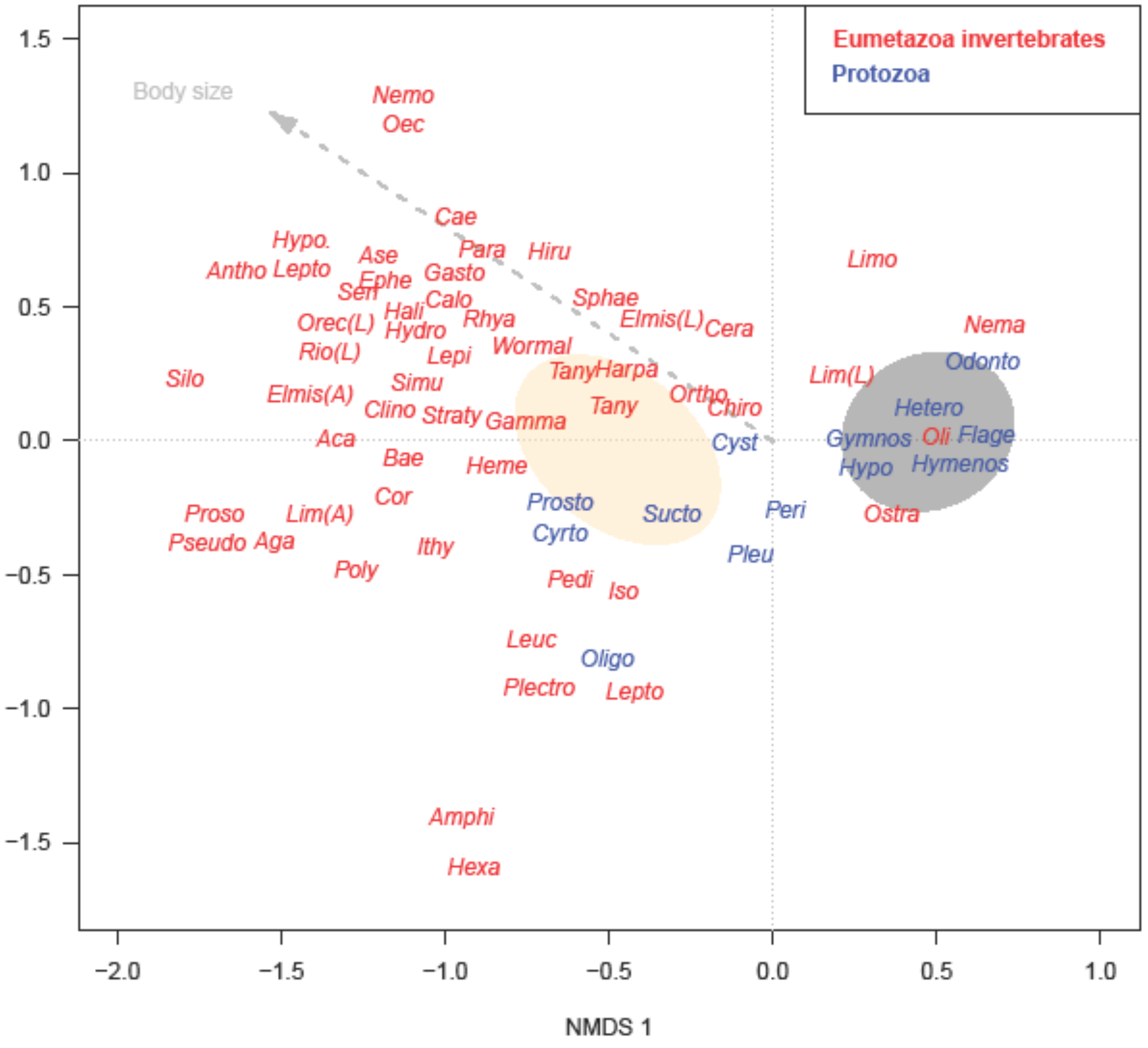

Fig S6. Influence of different taxa with the NMDS ordination model based on Bray-Curtis index comparing the dissimilarities in composition and abundance of benthos and hyporheos across the 30 studied systems. Ellipses show the 95% CIs on the location of centroids for benthos (yellow) and hyporheos (grey). Ellipses are kept in the panel to facilitate interpretation. The arrows depict the relationship of body size with the ordination. Code for taxa names is available in Table S2.

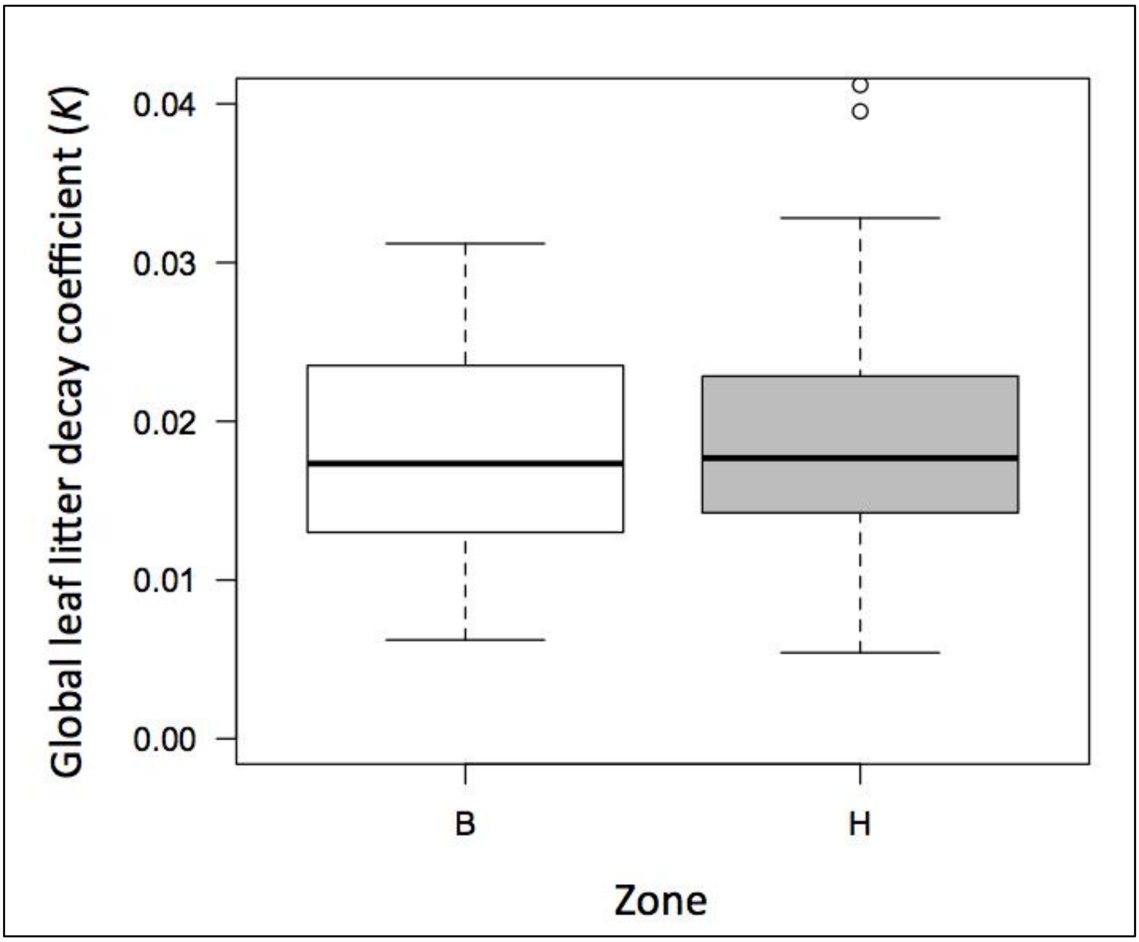

Fig S7. Descriptive Box-Plot showing global litter decay rate (*K*) values in the benthic and hyporheic zone of the studied systems.

Supplementary Methods.

Preparing and processing of cotton–strips and tea bags – Tensile strength of all cotton–strips was measured with an Instron Series IX tensiometer (Instron Corporation,Canton, Ohio) at 20 °C and 65% relative humidity in a climate–controlled room. Mean and standard deviation of pre–incubation tensile strength (631.0 ± 17 kg) was measured using 5 new cotton–strips. The green and rooibos–tea bags were dried for 2 days at 55°C and weighed (total bag weight) before incubation in the field. Initial bag weights were 2.12 g (SD = 0.02 g) and 2.15 g (SD = 0.02 g) for green–tea bags and rooibos–tea bags, respectively.

Samples preparation for organisms processing – Once in the laboratory, falcon vials containing bioassays collected in the field were shaken continuously for 1 min at 2500 rpm using a compact vortex shaker (SciQuip Vortex Mixers). Immediately after shaking, 10 ml water was collected with a pipette. From the collected water, 5 ml were filtered using cellulose acetate membrane filters (45 µm) to remove Protozoa and Eumetazoa invertebrates from the medium for later measurements of prokaryote biomass diversity and potential metabolic activity. The remaining water was kept unfiltered to process Protozoa. Both filtered and unfiltered water samples were stored in sterile conditions at 4 °C. The remaining content of the vials was retained on a 40–µm sieve for identification. Tea–bags and cotton–strips were stored and the remaining sieve contents were preserved in 4% formalin containing Bengal–rose stain so that invertebrates could be processed at a later time.

Body size-dry carbon content conversions – Body dimensions of all counted Protozoa and meiofauna (Eumetazoa invertebrates whose body size is into the range of 0.45–500.00 µm) were transformed to biovolume after Reiss and Schmid–Araya (2010). Protozoa individual biovolume was directly converted to dry carbon content assuming 0.14 pg C/μm^3 (Putt & Stoecker, 1996). For meiofauna individual biovolume was first converted into fresh mass using published gravity values (Feller & Warwik, 1998) following the approach of previous studies (i.e. Reiss & Schmid–Araya, 2008; Tod & Schmid–Araya, 2009; Peralta–Maraver, Reiss, & Robertson, 2018). Measurements of macroinvertebrates (Eumetazoa invertebrates whose body size is larger than 500.00 µm) were converted to dry mass using published body length and biovolume formula (Feller

& Warwick, 1998; Benke, Huryn, Smock, & Wallace,1999; Reiss & Schmid–Araya 2008; Tod & Schmid–Araya 2009). The individual carbon content of all Eumetazoa invertebrates was then calculated by using dry/wet mass ratio of 0.25 and dry mass/carbon content of 0.4 (Feller & Warwick 1998).

Ecoplates processing – EcoPlates had 96 wells containing 31 different dissolved carbon sources and a blank (a control well which contains only water), replicated three times. EcoPlate substrates were grouped into six categories according to Feigl, Ujaczki, Vaszita and Molnár (2017): carbohydrates, carboxilic acids, phenolic compounds, amino acids and polymers (grouping of substrates is available as Table S9).

Table S9. Carbon source categories grouping the BIOLOG EcoPlate substrates.

Carbon source category	Eco-plate substrate
Amino acids	• L- arginine<br>• L-asparagine<br>• L-phenylalanine<br>• L-serine<br>• glycyl-L-glutamic acid<br>• L-theronine
Carbohydrates	• D-mannitol, glucose-1-phosphate,<br>• D,L- alpha-glycerol phosphate,<br>• beta-methyl-D-glucoside,<br>• D-galactonic acid-gamma-lactone,<br>• i- erythritol,<br>• D-xylose,<br>• N-acetyl-D-glucosamine,<br>• D-cellobiose,<br>• alpha-D-lactose
Carboxylic acids	• D-glucosaminic acid<br>• D-malic acid<br>• itaconic acid<br>• pyruvic acid methyl ester<br>• D- galactouronic acid<br>• alpha-ketobutiryc acid<br>• gamma-hydroxybutyric acid
Phenolic compound	• 2-hydroxy benzoic acid<br>• 4-hydroxy benzoic acid
Amines	• phenylethylamine<br>• putrescine
Polymers	• Tween 40<br>• Tween 80<br>• alpha- cyclodextrine<br>• glycogen

A 100–µL aliquot was pipetted into each EcoPlate well and incubated in the dark at 15 °C for 5 days (three replicates by zone and study site by EcoPlate). After this period, colour development of each carbon category was measured as optical density (*OD*) at 595 nm using a Biotek HT absorbance reader (Biotek, Swindon, U.K.). Following EcoPlate protocols, *OD* values were corrected by subtracting the blank values in each EcoPlate and setting negative values to 0. Corrected *OD* values were used to calculate the plate average well colour development (*AWCD*) as:

$$(1)\ AWCD = \sum OD_i / N$$

where OD_i is the corrected *OD* value of each substrate containing well and *N* is the number of substrates (31) (Gryta, Frąc, & Oszust 2014). The average well colour development values for each substrate (*Substrate AWCD*) were also obtained using equation 2. For that propose, OD_i represented the corrected *OD* value of the substrates within the substrate category and *N* was the number of substrates in the category (Kenarova, Radeva, Traykov, & Boteva, 2014).

Prokaryotic functional diversity was calculated as Shannon–Wiener diversity value (*H´*) based on substrate utilization as:

$$(2)\ H' = -\sum_{\mathrm{i}=1}^{Sr} P_{\mathrm{i}} \ln(P_{\mathrm{i}})$$

where *Sr* is the number of wells with color development and P_i is the proportional colour development of the well over total color development of all wells of a plate.

Statistical analysis: models selection, fitting and models validation – Biomass of all identified groups and standardized decay coefficients (coefficients / standing stock biomass) were first Log_{10} transformed to solve heterogeneity of the residuals in the ANOVA tests and the regression models, but this was not necessary for the rest of the responses. Continuous covariates in the regression models were first centered by subtracting the mean and dividing by the standard deviation. Collinearity problems were detected among plate-AWCD (metabolic potential to utilize all carbon sources in EcoPlates) and individual substrate-AWCD (metabolic potential to utilize each carbon sources in EcoPlates) during data exploration. Therefore, plate-AWCD was maintained as a potential covariate of the regression models, while substrate-AWCD was not included. Intra–class correlation effects of the studied responses with the study site (samples collection from the same streams) and with catchment (streams sampled from the same catchment) were also detected during data exploration. Therefore, study site

(30 levels) and catchment (10 levels) were incorporated in the ANOVA tests as random factors (two random factors ANOVA) and the mediated regression models as random intercepts (Linear Mixed Models, LMMs).

In the case of mediated LMMs, backward selection based on the Akaike Information Criterion (AIC) was applied to find the optimal candidate for the mediation models. Full models containing all biological variables (mediators), streambed compartment and first–order interactions were fitted first. Then, less influential variables (lower AIC) were dropped sequentially using the drop1 function within R (R Core Team, 2014), and the reduced model was refitted at each time. Final mediation models were then fitted as part of the casual step method proposed by Judd and Kenny (1981) for statistical mediation analysis:

1) $Y = i_0 + cX + \varepsilon_0$

2.1) $= i_1 + a_1X + \varepsilon_1$

2.2) $M_2 = i_2 + a_2X + \varepsilon_2$

2.j) $M_j = i_j + a_jX + \varepsilon_j$

3) $Y = i_j + c'X + b_1M_1 + b_2M_2 + \ldots b_jM_j + \varepsilon_{j+1}$

where equation 1 shows the total effect of streambed compartment (X) on the response (Y). Equations 2.1 to 2.j show the effect of X on the mediator variables (M_1, M_2, …, M_j) included in the mediation model. Equation 3 (mediation model) shows the mediated effect of X (c') and the effect of the mediators ($b_{1,}$ $b_{1,\ldots}$ b_j) on Y. Interaction terms were added as the multiplication between streambed compartment and mediators in equation 3. In all equations, *i* represents the intercept and ε the error term. Proportion of the mediated effect was finally estimated as 1-(c'/c) (see Rijnhart, Twisk, Chinapaw, de Boer, & Heymans, 2017). According to the casual step method (Fig S3) the effect of exposure outcome variable (here streambed compartment) on the response (here decay coefficients) is mediated when a_1 to a_j, b_1 to b_j, and c coefficients are all significant (equations 1 and 2). Partial mediation occurs when c' is also significant, while full mediation occurs when c' coefficient is not significant. Following Rijnhart, Twisk, Chinapaw, de Boer and Heymans (2017), we reported both statistical significance and credible intervals for the coefficients estimation.

Previous equations were fitted using the function lmer of the R–package lme4 (Bates et al. 2016). Subsequently, 5000 values from the posterior joint distribution of the equation parameters were simulated with the function sim of the R–package arm

(Gelman & Su, 2009). This function uses an analytical direct-simulation method with uninformative priors (Gelman & Su, 2009). Obtained means of the simulated values from the joint posterior distribution of model parameters were used as estimates, and the 2.5% and 97.5% quantiles as lower and upper limits of 95% credible intervals. Furthermore, The anova function (based on F–statistic) and the summary function (based on t–statistic) from the R–package lmerTest (Kuznetsova, Brockhoff, & Christensen, 2015) were used to assess significance of ANOVA tests and LMMs coefficients, respectively. These functions implement the Kenward–Roger's method for approximating degrees of freedom. This is a robust method, which allows obtaining the p–values related with the F and t statistic when applying mixed effect models (Kuznetsova, Brockhoff, & Christensen, 2017). Finally, the marginal–R^2 (variance explained by fixed factors) and conditional–R^2 (variance explained by both fixed and random factors) of the models were calculated to assess model fit using the function rsquaredGLMM of the R–package MuMIn (Nakagawa & Schielzeth, 2013). Subsequently, partial R^2 of each variable included in the LMMs were assessed using the function r2beta of R–package r2glmm (Jaeger, 2016). Validation of underlying assumptions of normality and homoscedasticity in ANOVA tests and optimal mediation LMMs residuals was applied following Zuur, Ieno, Walker, Savaliev and Smith, (2009).

References

Bates, D., Maechler, M., Bolker, B., Walker, S., Christensen, R. H. B., & Singmann, H. (2013). Linear mixed-effects models using Eigen and S4. R package version 1.0-5. https://cran.r-project.org/web/packages/lme4

Benke, A. C., Huryn, A. D., Smock, L. A., & Wallace, J. B. (1999). Length–mass relationships for freshwater macroinvertebrates in North America with particular reference to the southeastern United States. *Journal of the North American Benthological Society*, 18, 308–343. doi: 10.2307/1468447

Gelman, A., & Su, Y. S. (2016). arm: Data analysis using regression and multilevel/hierarchical models. R package version 1.8-6. http://CRAN.R–project.org/package=arm

Feller, R. J., & Warwick, R. M. (1988) Energetics. In R. Higgins & H. Thiel (eds), *Introduction to the study of meiofauna* (pp. 181–196). Washington, DC: Smithsonian Institution Press.

Feigl, V., Ujaczki, É., Vaszita, E., & Molnár, M. (2017). Influence of red mud on soil microbial communities: Application and comprehensive evaluation of the Biolog EcoPlate approach as a tool in soil microbiological studies. *Science of the Total Environment*, 595, 903–911. doi: 10.1016/j.scitotenv.2017.03.266

Gryta, A., Frąc, M., & Oszust, K. (2014). The application of the Biolog EcoPlate approach in ecotoxicological evaluation of dairy sewage sludge. *Applied biochemistry and biotechnology*, 174(4), 1434–1443. doi: 10.1007/s12010-014-1131-8

Jaeger, B. (2016). R2glmm: Computes R squared for mixed (multilevel) models. R package version 0.1.1. https://cran.r-project.org/web/packages/r2glmm

Judd, C. M., & Kenny, D. A. (1981). Process analysis: Estimating mediation in treatment evaluations. *Evaluation review*, 5, 602-619. doi: 10.1177/0193841X8100500502

Rijnhart, J. J., Twisk, J. W., Chinapaw, M. J., de Boer, M. R., & Heymans, M. W. (2017). Comparison of methods for the analysis of relatively simple mediation models. *Contemporary clinical trials communications*, 7, 130-135. doi: 10.1016/j.conctc.2017.06.005

Kenarova, A., Radeva, G., Traykov, I., & Boteva, S. (2014). Community level physiological profiles of bacterial communities inhabiting uranium mining impacted sites. *Ecotoxicology and environmental safety*, 100, 226–232. doi: 10.1016/j.ecoenv.2013.11.012

Kuznetsova, A., Brockhoff, P. B., Christensen, R. H. B. (2015). lmerTest: Tests in Linear Mixed Effects Models. R Package Version 2.0–20. R Foundation for Statistical Computing, Vienna https://cran.r–project.org/web/packages/lmerTest/index.html.

Kuznetsova, A., Brockhoff, P. B., & Christensen, R. H. (2017). lmerTest package: Tests in linear mixed effects models. *Journal of Statistical Software*, 82(13), 1–26. doi: 10.18637/jss.v082.i13

Nakagawa, S., & Schielzeth, H. (2013). A general and simple method for obtaining R2 from generalized linear mixed-effects models. *Methods in Ecology and Evolution*, 4, 133–142. doi: https://doi.org/10.1111/j.2041-210x.2012.00261.x

Peralta–Maraver, I., Reiss, J., & Robertson, A. L. (2018). Interplay of hydrology, community ecology and pollutant attenuation in the hyporheic zone. Science of The Total Environment, 610, 267–275. doi: 10.1016/j.scitotenv.2017.08.036

Putt, M., & Stoecker, D. K. (1989). An experimentally determined carbon: volume ratio for marine "oligotrichous" ciliates from estuarine and coastal waters. *Limnology and Oceanography*, 34(6), 1097–1103. doi: 10.4319/lo.1989.34.6.1097

R Core Team (2014). R: A language and environment for statistical computing. R Foundation for Statistical Computing, Vienna, Austria. Retrieved from http://www.R–project.org/

Reiss, J., & Schmid–Araya, J. M. (2008). Existing in plenty: abundance, biomass and diversity of ciliates and meiofauna in small streams. *Freshwater Biology*, 53(4), 652–668. doi: 10.1111/j.1365-2427.2007.01907.x

Reiss, J., & Schmid–Araya, J. M. (2010). Life history allometries and production of small fauna. *Ecology*, 91(2), 497–507. doi: 10.1890/08-1248.1

Tod, S. P., & Schmid–Araya, J. M. (2009). Meiofauna versus macrofauna: secondary production of invertebrates in a lowland chalk stream. *Limnology and Oceanography*, 54, 450–456. Doi: 10.4319/lo.2009.54.2.0450

Zuur, A. F., Ieno, E. N., Walker, N. J., Savaliev, A. A. & Smith, G. M. (2009). *Mixed Effects Models and Extensions in Ecology with R*. New York: Springer,
